# Rapid sequential clustering of NMDARs, CaMKII, and AMPARs upon activation of NMDARs at developing synapses

**DOI:** 10.3389/fnsyn.2024.1291262

**Published:** 2024-04-10

**Authors:** Yucui Chen, Shangming Liu, Ariel A. Jacobi, Grace Jeng, Jason D. Ulrich, Ivar S. Stein, Tommaso Patriarchi, Johannes W. Hell

**Affiliations:** ^1^Department of Pharmacology, University of Iowa, Iowa City, IA, United States; ^2^Department of Pharmacology, University of California, Davis, Davis, CA, United States

**Keywords:** AMPA receptor (AMPAR), calcium, calmodulin (CAM), CaMKII, synapse, NMDA receptor (NMDAR)

## Abstract

Rapid, synapse-specific neurotransmission requires the precise alignment of presynaptic neurotransmitter release and postsynaptic receptors. How postsynaptic glutamate receptor accumulation is induced during maturation is not well understood. We find that in cultures of dissociated hippocampal neurons at 11 days *in vitro* (DIV) numerous synaptic contacts already exhibit pronounced accumulations of the pre- and postsynaptic markers synaptotagmin, synaptophysin, synapsin, bassoon, VGluT1, PSD-95, and Shank. The presence of an initial set of AMPARs and NMDARs is indicated by miniature excitatory postsynaptic currents (mEPSCs). However, AMPAR and NMDAR immunostainings reveal rather smooth distributions throughout dendrites and synaptic enrichment is not obvious. We found that brief periods of Ca^2+^ influx through NMDARs induced a surprisingly rapid accumulation of NMDARs within 1 min, followed by accumulation of CaMKII and then AMPARs within 2–5 min. Postsynaptic clustering of NMDARs and AMPARs was paralleled by an increase in their mEPSC amplitudes. A peptide that blocked the interaction of NMDAR subunits with PSD-95 prevented the NMDAR clustering. NMDAR clustering persisted for 3 days indicating that brief periods of elevated glutamate fosters permanent accumulation of NMDARs at postsynaptic sites in maturing synapses. These data support the model that strong glutamatergic stimulation of immature glutamatergic synapses results in a fast and substantial increase in postsynaptic NMDAR content that required NMDAR binding to PSD-95 or its homologues and is followed by recruitment of CaMKII and subsequently AMPARs.

## Introduction

Presynaptic release of glutamate, the main neurotransmitter in the brain, rapidly activates postsynaptic AMPARs that are precisely juxtaposed to the release sites (MacGillavry et al., 2013; Nair et al., 2013; Tang et al., 2016). Most AMPARs in the developed forebrain consist of two GluA2 plus either two GluA1 or two GluA3 subunits and conduct Na^+^ (Lu et al., 2009). Heightened synaptic activity leads to activation of NMDARs, which conduct Ca^2+^ and are mainly formed by two GluN1 plus two GluN2A or GluN2B subunits in the mature forebrain (Sheng et al., 1994; Wenthold et al., 2003). AMPARs and NMDARs are anchored at postsynaptic sites by PSD-95 and its homologs PSD-93 and SAP102 (Schnell et al., 2002; Ehrlich and Malinow, 2004; Schluter et al., 2006; Elias et al., 2008; Hafner et al., 2015; Matt et al., 2018; Buonarati et al., 2019). Their simultaneous knock down drastically reduces excitatory postsynaptic currents by AMPARs and NMDARs and the number of dendritic spines with large heads without affecting density of more immature thin spines (Elias et al., 2008; Levy et al., 2015). These data suggest that substantial enrichment of AMPAR and NMDAR occurs late during synapse maturation. NMDARs accumulate first to form so-called silent synapses before AMPARs are recruited upon NMDAR activation (Isaac et al., 1995; Gray et al., 2011). Furthermore, Ca^2+^ influx via NMDARs is usually critical for long-term potentiation (LTP) (Malenka and Bear, 2004; Huganir and Nicoll, 2013; Buonarati et al., 2019), and LTP or LTP-like mechanisms constitute an essential part of synapse development (Isaac et al., 1995; Sanhueza et al., 2011; Incontro et al., 2018). Remarkably, when neurotransmitter release is abolished, synaptic structures that include presynaptic terminals with synaptic vesicles and dendritic spines with postsynaptic densities still form but these immature synapses do not acquire functionally detectable NMDARs and AMPARs (Verhage et al., 2000; Varoqueaux et al., 2002; Sando et al., 2017; Sigler et al., 2017).

AMPARs and likely NMDARs are inserted via recycling endosomes into the plasma membrane outside postsynaptic sites (Gerges et al., 2004; Park et al., 2004, 2006; Bowen et al., 2017; Buonarati et al., 2019). Glutamate receptors reach postsynaptic sites by lateral diffusion where they are trapped by interactions with PSD-95 and its homologs (Groc et al., 2006; Bats et al., 2007; Bard et al., 2010; Penn et al., 2017; Patriarchi et al., 2018). For their postsynaptic anchoring, PSD-95 binds directly to NMDAR GluN2 subunits (Bard et al., 2010) and to auxiliary AMPAR subunits known as transmembrane AMPAR regulatory proteins (TARPs), including stargazin/γ_2_ and its homologs γ_3_, γ_4_, and γ_8_ (Chen et al., 2000; El-Husseini et al., 2000; Schnell et al., 2002; Patriarchi et al., 2018; Buonarati et al., 2019).

In young primary hippocampal cultures (7-11 DIV), repetitive depolarization with KCl induces exocytosis of synaptic vesicles at already existing presynaptic boutons, identified by colocalization of presynaptic markers with postsynaptic PSD-95 puncta (Yao et al., 2006) (see also Kraszewski et al., 1995; Krueger et al., 2003). This repetitive depolarization with KCl leads to an increase in amplitude and frequency of mEPSCs in postsynaptic neurons in young (7-11 DIV) but not mature (18-22 DIV) cultures (Yao et al., 2006). That increase in mEPSC amplitude but not the increase in frequency requires postsynaptic glutamate receptor activation, likely induced by KCl-mediated presynaptic depolarization leading to glutamate release (Yao et al., 2006). This separation of presynaptic (increase in mEPSC frequency) and postsynaptic modulation (increase in mEPSC amplitude) suggests that repetitive depolarization triggers an increase in functionally available glutamate receptors at postsynaptic sites. In support of this interpretation, activation of NMDARs by glutamate application induces a fast increase in synaptic GluA1 puncta and in EPSC amplitude in hippocampal cultures within 5 min (Antonova et al., 2001; Wang et al., 2005). Furthermore, glutamate release due to spontaneous activity from defined presynaptic sites in hippocampal cultures is sufficient to trigger GluA1 accumulation on juxtaposed postsynaptic sites by trapping of GluA1 undergoing lateral diffusion (Ehlers et al., 2007). However, how postsynaptic accumulation of NMDARs is induced during synaptogenesis in immature neurons is unknown.

Instructive transsynaptic signaling by presynaptic glutamate release would constitute a simple model for the coordination of presynaptic and postsynaptic development. We tested whether brief glutamate applications induce postsynaptic accumulation of NMDARs analogous to the previously observed glutamate-induced AMPAR accumulation (Antonova et al., 2001; Wang et al., 2005). We report that glutamate induced an unexpected rapid and prolonged clustering of NMDARs. Our primary immature 11 DIV hippocampal cultures contained numerous synaptic structures defined by clusters that were immunoreactive for different pre- and postsynaptic marker proteins. These puncta showed only modest glutamate receptor immunoreactivity that was not above immunosignals from surrounding areas. Numerous prominent GluN1 and GluN2A clusters became prominent within 1 min of glutamate or NMDA treatment. These clusters precisely colocalized with pre-and postsynaptic markers. The density of NMDAR clusters remained increased for at least 3 days following 1 min glutamate application. This increase in NMDAR clusters was paralleled by a lasting increase in NMDAR mEPSC amplitude. NMDAR clustering was followed by CaMKII clustering within 3 min and AMPAR clustering within 6 min. This sequel is especially remarkable in the light of recent findings that full maturation of synapses to their full strength of AMPAR activity requires binding of catalytically active CaMKII to the NMDAR (Incontro et al., 2018; Tao et al., 2021).

## Results

### Glutamate treatment induces rapid postsynaptic NMDAR clustering

**FIGURE 1:**
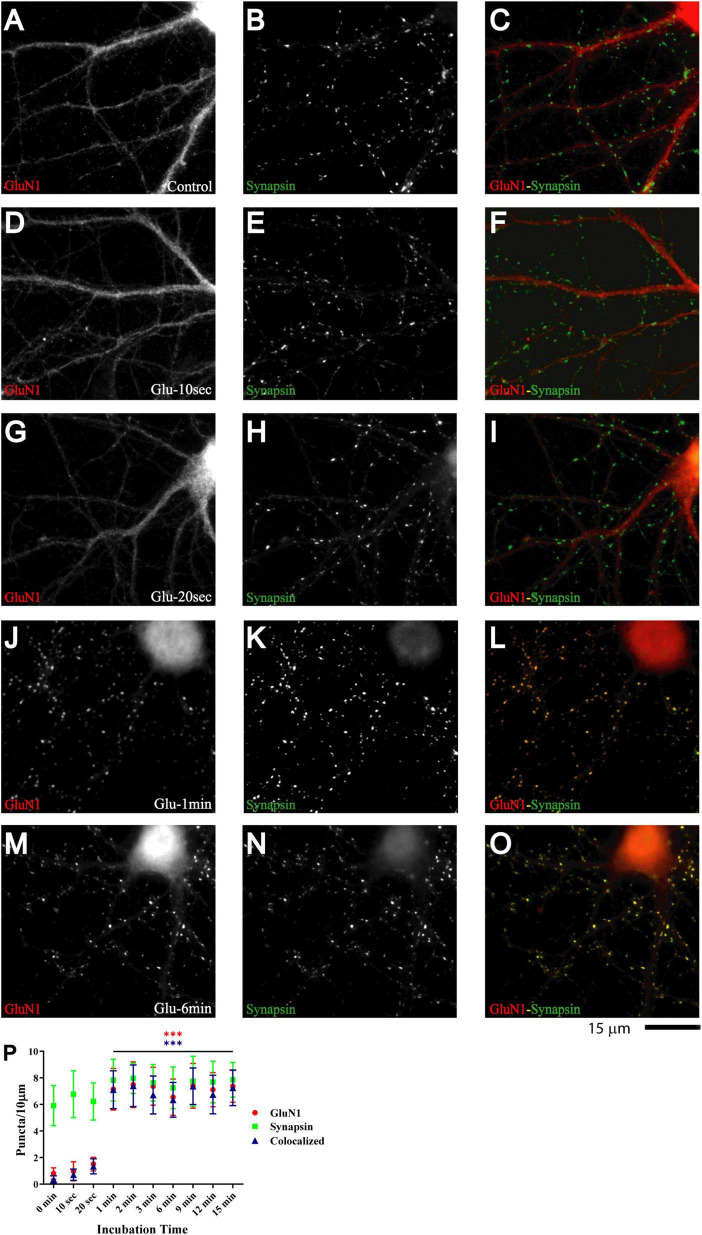
Glutamate induces rapid NMDAR redistribution in 11 DIV hippocampal neurons. Immunofluorescence images of 11 DIV hippocampal cultures (HC) following treatment with vehicle for 1 min **(A–C)** or 100 μM glutamate for 10 s **(D–F)**, 20 s **(G–I)**, 1 min **(J–L)**, and 6 min **(M–O)** and subsequent immunostaining for GluN1 and synapsin. GluN1 is smoothly distributed throughout dendrites and cell bodies under control conditions and following short glutamate applications (left panels; red in overlay in right panels). Synapsin has a punctate appearance along dendritic shafts under all conditions (middle panels; green in overlay in right panels). Quantification of cluster numbers for GluN1 shows a nearly tenfold increase in discernable puncta upon 1 min glutamate treatment (red circles in panel **P**). Nearly all GluN1 puncta after but not before glutamate treatment are colocalized with synapsin puncta (blue triangles in panel **P**) indicating that most of the newly formed but not preexisting GluN1 clusters are synaptic. Similar results were obtained for GluN2A and its colocalization with bassoon (Supplementary Figure 1).

Primary hippocampal cultures at 7-11 DIV are model systems of immature neurons and synaptogenesis [e.g., (Kraszewski et al., 1995; Ziv and Smith, 1996; Rao et al., 1998; Liao et al., 1999; Friedman et al., 2000; Krueger et al., 2003; Gerrow et al., 2006; Yao et al., 2006; Branco et al., 2008; Letellier et al., 2019)]. Glutamate application augments the number of synaptic GluA1 puncta and EPSC amplitude in such cultures within 5 min (Antonova et al., 2001; Wang et al., 2005). Similarly, repeated depolarization with KCl leads to an increase in amplitude of mEPSC, which requires postsynaptic glutamate receptor activation (Yao et al., 2006). To test whether glutamate also regulates postsynaptic NMDAR clustering, we applied 100 μM glutamate to 11 DIV primary hippocampal cultures. Cultures were fixed immediately after treatment, permeabilized, and stained for GluN1, which is present in all NMDARs. Under control conditions and after mock treatment with vehicle only, GluN1 largely showed a smooth distribution throughout dendrites and cell bodies. GluN1 underwent a striking redistribution upon treatment with glutamate for 1 min but not 20 s (Figure 1; compare G and J). The number of distinct immunoreactive GluN1 puncta in individual microscopic fields, which typically covered about 25% of the neuronal arborization, was minimal under control conditions and mostly not colocalized with other synaptic proteins but increased to ∼250 during the 1 min glutamate treatment (Figure 1P, red circles). A similar redistribution was observed for GluN2A (Supplementary Figure 1). We note that this dramatic increase in immunoreactive GluN1 and GluN2A puncta is a reflection of NMDAR staining reaching the threshold for detection as distinct puncta without providing a precise measure for the increase in the NMDAR number per synapse (but see below for more accurate quantitative analysis by mEPSC analysis).

**FIGURE 2:**
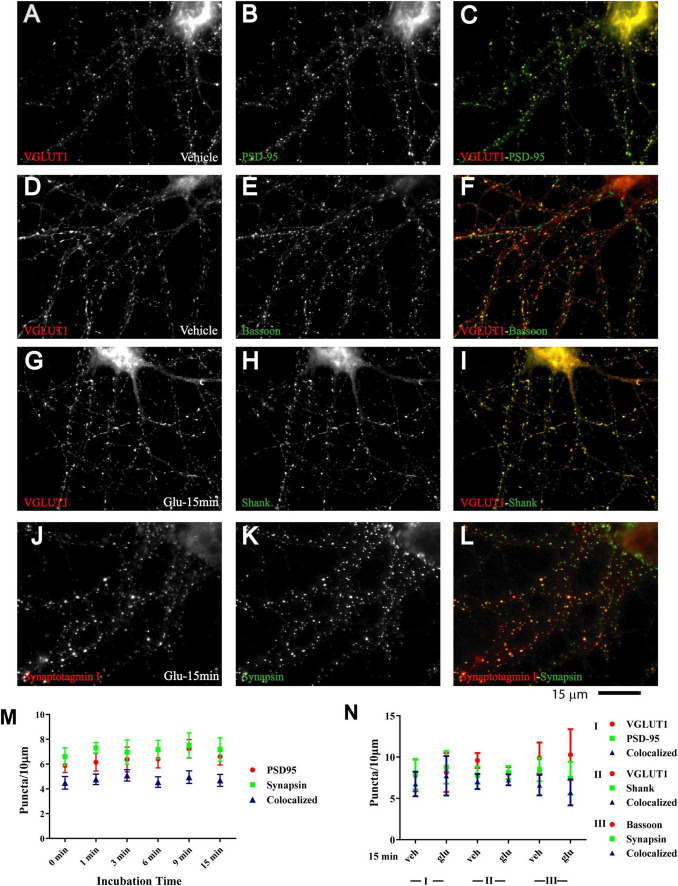
Immature synaptic structures in 11 DIV hippocampal neurons. Immunofluorescence images of 11 DIV HC following treatment with vehicle **(A–F)** or 100 μM glutamate for 15 min **(G–L)**. Existing synaptic contacts contain the vesicular glutamate transporter VGluT1 **(A,D,G)**, the presynaptic cytomatrix protein bassoon **(E)**, the synaptic vesicle-associated proteins synaptotagamin **(J)** and synapsin **(K)**, and the postsynaptic proteins PSD-95 **(B)** and Shank **(H)**. Quantification of cluster number and of their colocalization indicates that distribution of these synaptic proteins is not altered by prolonged glutamate treatment **(M,N)**.

Nearly all newly formed GluN1 puncta were colocalized with synapsin and GluN2A puncta with bassoon (Figure 1 and Supplementary Figure 1). Synapsin is a membrane-associated synaptic vesicle protein and bassoon a component of the presynaptic cytomatrix. Both are well-established markers for nerve terminals. To further evaluate whether these synapsin- and bassoon-positive puncta are indicative of synaptic structures, we analyzed the distribution of other synaptic markers. All showed a punctate distribution in the 11DIV hippocampal cultures and colocalized with each other already under basal conditions without glutamate treatment. These proteins included the prevalent glutamate transporter of synaptic vesicles VGluT1, the synaptic vesicles proteins synaptotagmin and synaptophysin, and PSD-95 and Shank, two central structural components of the postsynaptic site (Figure 2 and below). The mainly smooth distribution of GluN1and GluN2A in unstimulated 11DIV neurons indicates that NMDA receptors are present at these synaptic sites but not strongly enriched as would be expected for fully mature synapses (Figure 1A and below). Accordingly, the synaptic contacts defined by co-clusters of pre- and postsynaptic markers at 11DIV contain a substantially lower level of NMDARs than fully mature synapses although all other so far analyzed synaptic proteins are already highly concentrated at these puncta. A similarly low level of synaptic accumulation was also observed for AMPARs in our unstimulated cultures (see below). The synaptic structures defined by co-clusters of synapsin, synaptophysin, synaptotagmin, VGluT1, bassoon, PSD-95, and Shank under basal conditions at 11 DIV were thus not completely mature synapses as they lack the full content of glutamate receptors. However, presynaptic terminals underwent spontaneous exocytosis/endocytosis cycles of synaptic vesicles as indicated by anti-synaptotagmin antibody uptake (Supplementary Figure 2). Glutamate induced fast GluN1and GluN2A clustering without altering the distribution of the aforementioned synaptic markers (Figure 2).

A quantitative analysis indicates that the vast majority of glutamate-induced GluN1 and GluN2A clusters colocalized with synapsin and bassoon, respectively (Figure 1P and Supplementary Figure 1 blue triangles). Furthermore, most synapsin and bassoon puncta that were indicative of immature synaptic contacts in our 11DIV cultures were enriched for GluN1 and GluN2A immunoreactivity after glutamate treatment. These findings suggest that most of these synaptic contacts rapidly accumulated NMDARs during the treatment. This accumulation likely reflects clustering of NMDARs at the surface of postsynaptic sites. To directly address this issue, we performed surface labeling with the GluN1 antibody, which is directed against an extracellular epitope of the GluN1 subunit. Although GuN1 surface labeling is much more difficult to accomplish, it is obvious that glutamate treatment led to formation of puncta that overlapped with PSD-95 puncta (Supplementary Figure 3). Because GuN1 surface labeling is limited, we also used imaging of GluN1 tagged with superecliptic pHluorine (SEP), which only fluoresce when exposed to a neutral pH and not inside acidic secretory or endosomal vesicles. As seen for immunostainings of endogenous NMDARs, 1 and 3 min but not 30 s long treatments with glutamate induced clustering of SEP-GluN1 (Supplementary Figure 4). These results indicate that the glutamate-induced NMDAR clustering occurs at the surface of postsynaptic sites.

We conclude that at 11 DIV hippocampal neurons possess synaptic contacts that are partially functional as they undergo exocytosis and endocytosis of synaptic vesicles but lack the full complement of AMPARs and NMDARs of mature synapses (see also mEPSC analysis below). We further conclude that brief glutamate application induces accumulation of NMDARs thereby advancing synapse maturation.

### Glutamate-induced NMDAR clustering is long-lasting

**FIGURE 3:**
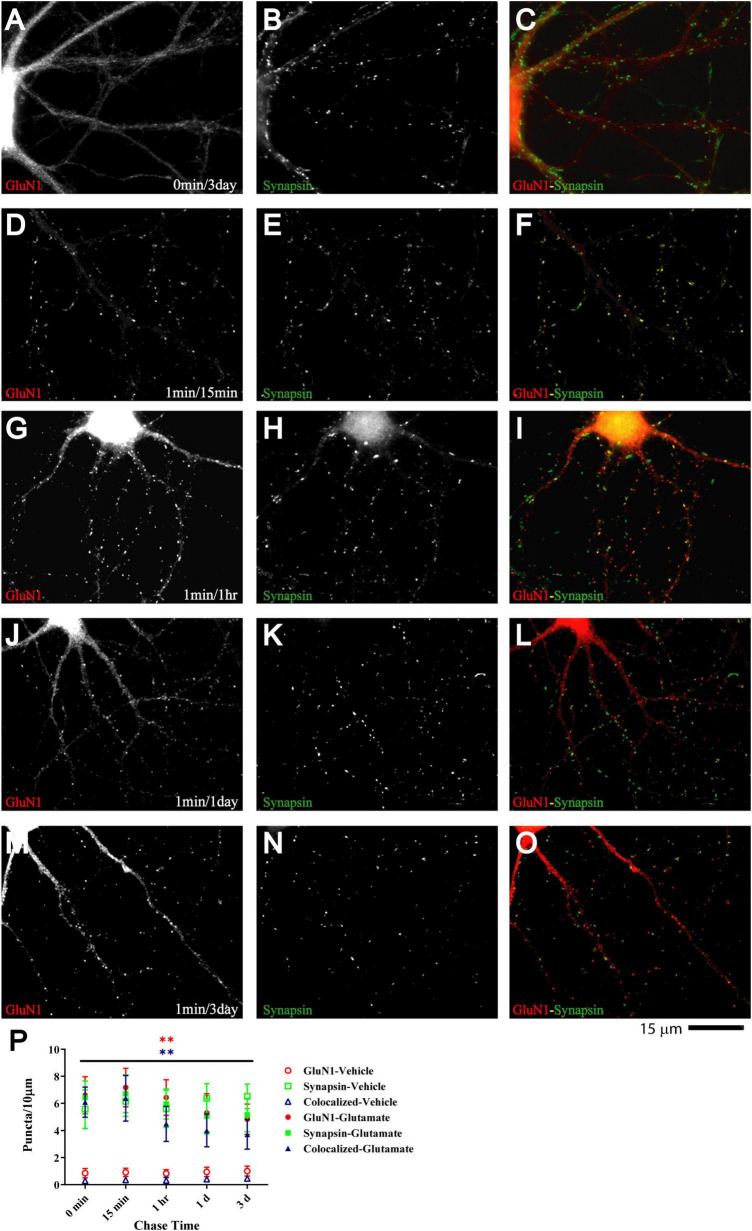
Glutamate-induced NMDAR clustering is persistent. 11 DIV hippocampal cultures were treated with vehicle **(A–C)** or 100 μM glutamate for 1 min before chased in glutamate-free medium for 15 min **(D–F)**, 1 h **(G–I)**, 1 d **(J–L)**, and 3 d (**A–C** and **M–O**). Glutamate (but not vehicle; **A**) induced GluN1 clustering (left panels) that is still obvious after 3 d **(M)**. Synapsin distribution remains unaltered (middle panels). Quantification indicates that the number of glutamate-induced total GluN1 clusters (filled red circles) and their degree of colocalization with synapsin puncta (filled blue triangles) is increased after 1 min glutamate treatment without a chase (0 min time point in panel **P**) and remains significantly elevated for 3 days **(P)**. Control samples that underwent 1 min treatment with vehicle (water) showed no increase in GluN1 cluster density (open red circles and blue triangles) during subsequent 1–3 d chase periods. Accordingly, very few if any synapse matured between 11 and 14 DIV (but see Supplementary Figure 5, which illustrates an increase in GluN1 cluster density between 15 and 18 DIV).

If glutamate-triggered NMDAR clustering contributes to synaptic maturation, it should be long-lasting. To test whether this clustering is stable, cultures were treated with glutamate for 1 min before chase periods of increasing length. The glutamate-induced increase in clustering remained prominent over the whole time course tested, which included 15 min, 1 h, 1 day, and 3 days (Figure 3). Mock treatment with vehicle at 11 DIV followed by a 3 day chase showed no increase in cluster number of GluN1 (Figure 3A; open red circles and open blue triangles in P) or GluN2A (data not illustrated). Glutamate-induced GluN1 clusters were largely maintained over time; it appears that there might be a modest decline in the number of clusters seen after a 1 and a 3 day chase versus the earlier time points but this decline did not reach statistical significance (Figure 3P, filled red circles and filled blue triangles). Note that in our unstimulated cultures NMDAR clustering does not become prominent before 15–16 DIV (Supplementary Figure 5). Thus, the GluN1 clusters seen after 1 min glutamate treatment followed by a 3 day chase is not due to a developmentally-caused increase in GluN1 accumulation but because the glutamate-induced clustering is maintained. Accordingly, cultures at 11 DIV are well-suited for analyzing the final maturation steps of synapses.

### Glutamate-induced NMDAR clustering is paralleled by increased NMDAR mEPSC amplitude

**FIGURE 4:**
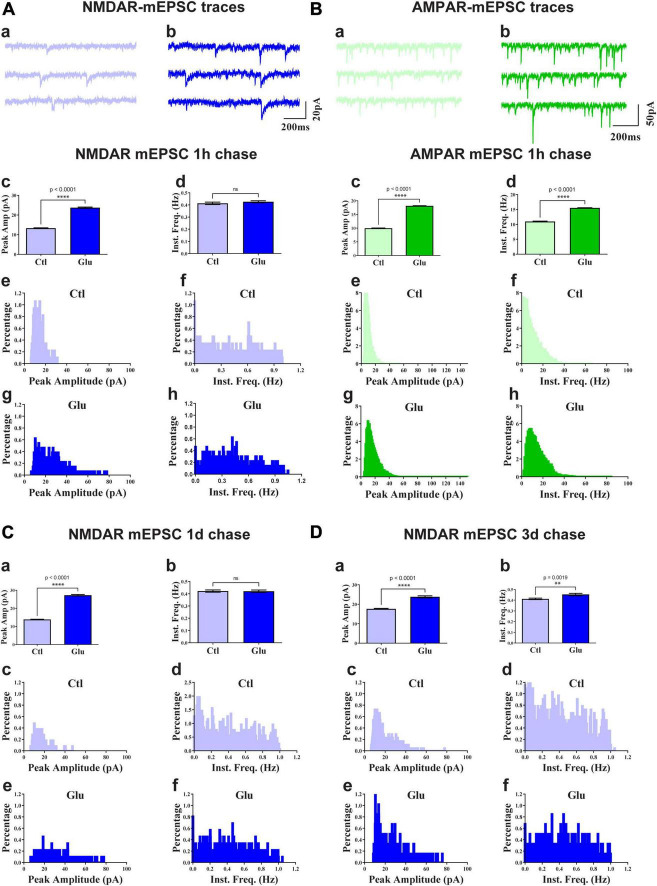
Glutamate increases NMDAR and AMPAR mEPSC amplitude. 11 DIV hippocampal cultures were treated with vehicle (Ctl) or 100 μM glutamate (Glu) for 1 min **(A,C,D)** or 6 min **(B)** and incubated for 1 h **(A,B)**, 1 d **(C)**, or 3 d **(D)** before mEPSCs were recorded for NMDAR **(A,C,D)** and AMPAR **(B)** before (a) and after (b) glutamate treatment and chase. Top panels show sample traces after 1 h chases for NMDARs **(A)** and AMPARs **(B)**. Other panels show bar diagrams and histograms of peak amplitudes and frequencies, as indicated. Compared to the respective vehicle control conditions, following 1 min glutamate treatment, the average peak amplitude but not instant frequency (Inst. Freq.) of NMDAR mEPSCs is increased by about 100% after chases for 1 h **(A)** and 1 d **(C)** and somewhat less after a 3 d chase (**D**; but to a similar final level as for 1 h and 1 d chases). The NMDAR mEPSCs amplitude is modestly increased by about 30% following control treatment between 11 and 14 DIV (compare 3 day chase in panel **D** with 1 h chase in panel **A**). The average peak amplitude and frequency of AMPA receptor mEPSCs is augmented after 1 h following 6 min glutamate treatment **(B)**.

To test whether glutamate-induced NMDAR clustering reflects an increase in functional NMDAR channels at the postsynaptic site we monitored mEPSCs from cultures treated for 1 min with glutamate followed by chase periods of 1h, 1d, and 3d. AMPAR were blocked with CNQX to obtain NMDAR-mediated responses. Glutamate treatment nearly doubled the amplitude of NMDAR mEPSCs but had minimal effect on mEPSC frequency (Figure 4). This increase in NMDAR current amplitude was maintained for at least 3 days (Figure 4). There was a slight increase in NMDAR mEPSCs amplitude under control conditions between 11 and 14 DIV (Figure 4; compare 3 day chase in D with 1 h chase in A) but the increase following NMDAR activation remained statistically highly significant. Thus, the observed stable increase in glutamate-induced synaptic NMDAR staining was paralleled by increased postsynaptic NMDAR responses. Collectively these observations indicate that glutamate promoted the accumulation of functional NMDAR at the postsynaptic surfaces. We conclude that brief application of glutamate to young neuronal cultures induces an unexpected rapid and stable accumulation of functional NMDAR at postsynaptic sites of immature synapses.

### Glutamate triggers fast CaMKII clustering subsequent to NMDAR clustering

**FIGURE 5:**
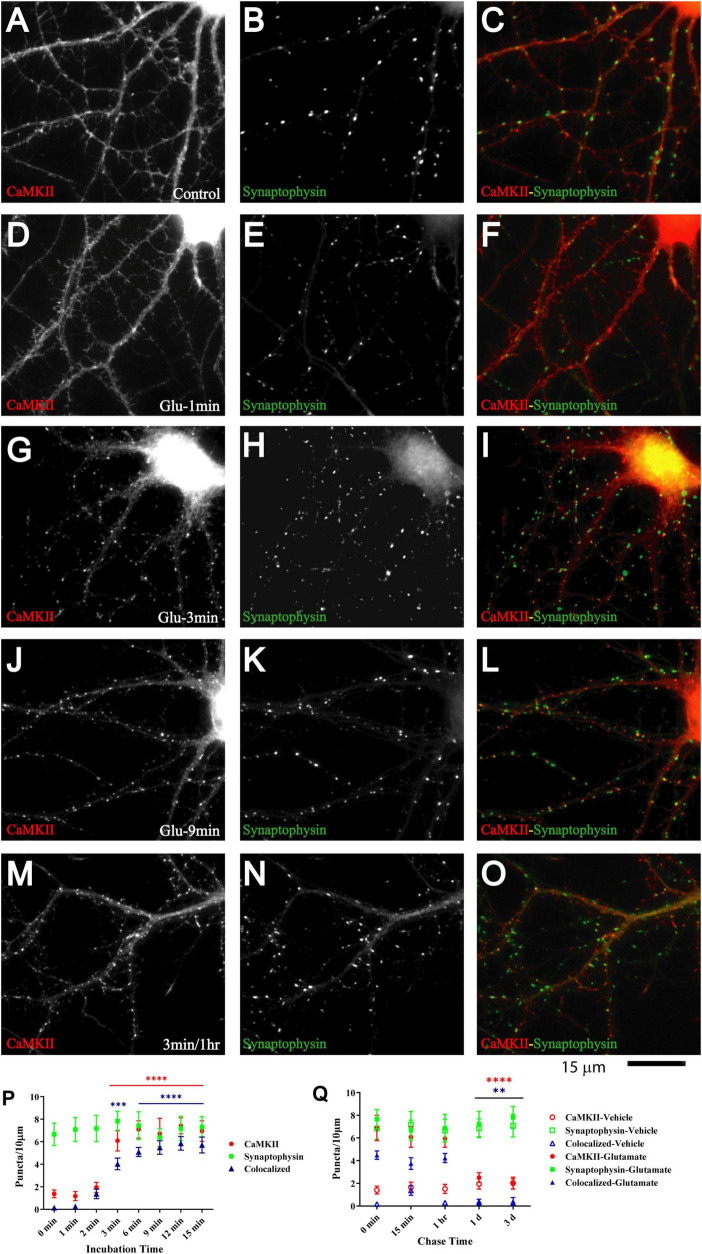
Glutamate triggers synaptic CaMKII clustering subsequent to GluN1 clustering. 11 DIV hippocampal cultures were treated with vehicle (control; **A–C**) or 100 μM glutamate for 1 min **(D–F)**, 3 min **(G–I, M–O)**, 9 min **(J–L)**, and 1 min and fixed either immediately after these treatments or following a 1 h chase **(M–O)**. Glutamate induced CaMKIIα clustering (left panels) within 3 but not 1 min, which lasts at least 1 h. Synaptophysin distribution remains unaltered (middle panels). Quantification indicates that the total number of CaMKIIα puncta is increased by three- to fivefold after 3–15 min glutamate treatment (red circles in panel **P**). The majority of the newly formed CaMKIIα puncta is colocalized with synapsin (blue triangles in panel **P**) indicating that they are synaptic, which was not the case for the CaMKIIα puncta observed under control conditions. Of note, virtually all CaMKIIα puncta detected under control conditions were not colocalized with synapsin whereas the majority of the newly formed CaMKIIα puncta is colocalized with synapsin (blue triangles in panel **P**) indicating that most newly formed CaMKIIα clusters are synaptic whereas nearly none of the CaMKIIα clusters detectable under control conditions or following a 1–3 min chase after glutamate treatment are colocalized with synapsin. Glutamate-induced CaMKIIα clustering is stable for at least 1 h but largely dispersed after 1 d when the puncta density is no longer statistically different from vehicle-treated controls (**Q**; filled red circles: total CaMKII puncta; filled blue triangles: CaMKII puncta colocalized with synapsin; note that the values for the CaMKII cluster densities following glutamate treatments overlap with respective cluster densities following vehicle control treatment for the 1 and 3 d chases (open symbols) so that some open symbols are hidden behind filled symbols).

The Ca^2+^- and calmodulin-dependent protein kinase CaMKII plays a critical role in synapse formation (Fink et al., 2003) and specifically AMPAR recruitment during synaptogenesis (Wu et al., 1996; Poncer et al., 2002; Incontro et al., 2018), in homeostasis of excitatory input into neurons (Thiagarajan et al., 2002; Pratt et al., 2003), LTP (Lledo et al., 1995; Malinow and Malenka, 2002; Collingridge et al., 2004; Malenka and Bear, 2004), and in learning and memory (Giese et al., 1998; Elgersma et al., 2002; Lisman et al., 2002) [reviewed in Hell (2014), Yasuda et al. (2022)]. CaMKII is directly associated with the NMDAR via at least three binding sites (Gardoni et al., 1998; Strack and Colbran, 1998; Leonard et al., 1999; Bayer et al., 2001; Merrill et al., 2007). Its binding to the NMDAR and its accumulation at postsynaptic sites depends on its activation by Ca^2+^ influx through the NMDAR (Strack and Colbran, 1998; Leonard et al., 1999; Shen and Meyer, 1999; Shen et al., 2000; Bayer et al., 2001; Colbran and Brown, 2004; Merrill et al., 2005). Furthermore, 1 h treatment of young neuronal cultures with aggregated ephrinB1-Fc to activate EphBs induces co-clustering of NMDARs with CaMKII (Dalva et al., 2000). Finally, binding of CaMKII to NMDAR is important for recruitment of AMPARs to the postsynaptic site (Sanhueza et al., 2011; Incontro et al., 2018). Therefore, we tested whether glutamate induces co-clustering of CaMKII with NMDARs. Induction of CaMKII clustering was detectable after 2 min and prominent after 3 min of glutamate treatment (Figure 5). CaMKII clustering remained enhanced in cultures treated with glutamate for 3 min during 1 h of chase as compared to vehicle-treated cultures (Figure 5M; open red circles and open blue triangles in Figure 5Q). However, CaMKII clustering was not maintained for 1 or 3 days (Figure 5Q). Together with the earlier evidence for a role for CaMKII in synapse development and plasticity, this behavior is consistent with a critical role for CaMKII during certain (i.e., early) periods of synapse maturation.

### Glutamate induces fast AMPAR clustering subsequent to clustering of NMDARs and CaMKII

**FIGURE 6:**
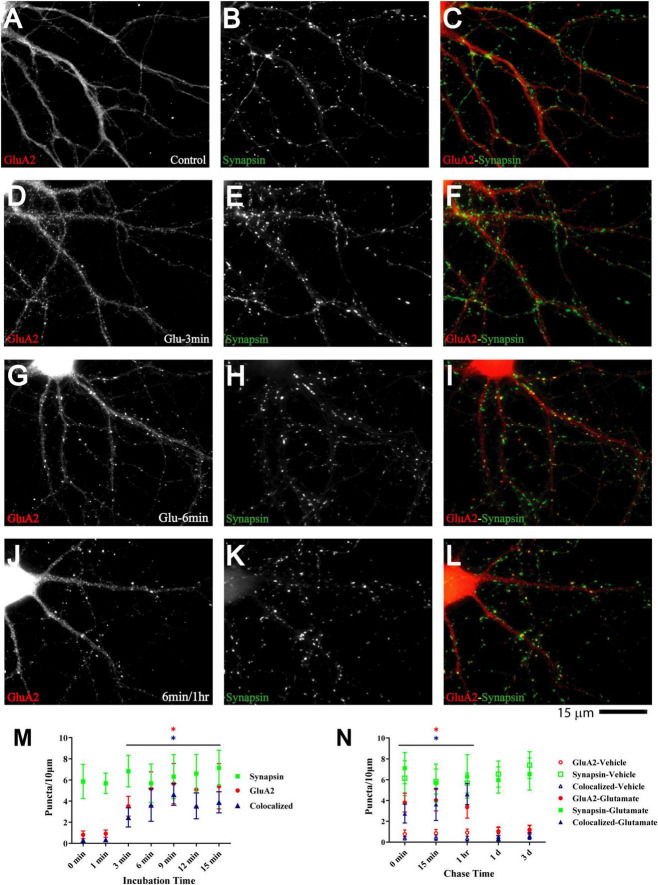
Glutamate induces GluA2 clustering subsequent to GluN1 and CaMKII clustering. 11 DIV hippocampal cultures were treated with vehicle (Control; **A–C**) or 100 μM glutamate for 3 min **(D–F)**, 6 min **(G–I)**, and 6 min followed by a 1 h chase **(J–L)** before fixation and staining. GluR2 accumulation is strongly increased by glutamate treatment for 6 min or longer (left panels) and lasts at least 1 h. Synapsin distribution remains unaltered (middle panels). Quantification indicates that the number of GluA2 puncta is increased by several fold after 6–15 min glutamate treatment (red circles in panel **M**). The majority of the newly formed GluA2 puncta is colocalized with synapsin (blue triangles in panel **M**) indicating that they are synaptic, which was not the case for the GluA2 puncta observed under control conditions. Glutamate-induced GluA2 clustering is stable for at least 1 h but largely dispersed after 1 d (**N**; filled red circles: total GluA2 puncta; filled blue triangles: GluA2 puncta colocalized with synapsin; note that the values for the AMPAR cluster densities following glutamate treatments overlap with respective cluster densities following vehicle control treatment for the 1 d and 3 d chases (open symbols) so that some open symbols are hidden behind filled symbols).

Glutamate-induced NMDAR and CaMKII clustering within 1–3 min in our system precedes the glutamate-induced GluA1 clustering described earlier, which takes 5–6 min (Antonova et al., 2001; Wang et al., 2005). Such a time course of events would support other findings that implicate NMDARs (Liao et al., 1995, 2001; Gomperts et al., 1998; Petralia et al., 1999), CaMKII (Wu et al., 1996; Poncer et al., 2002), and their interaction (Sanhueza et al., 2011; Incontro et al., 2018) (see also (Tao et al., 2021)) in postsynaptic AMPAR accumulation during synapse development. Similarly, Ca^2+^ influx through NMDARs and subsequent activation of CaMKII are critical for LTP (Malenka et al., 1989; Malinow et al., 1989; Hell, 2014; Yasuda et al., 2022), which is at least in part due to the increase in the number of functional AMPARs that are present at postsynaptic sites (Malinow and Malenka, 2002; Collingridge et al., 2004; Malenka and Bear, 2004; Lisman and Raghavachari, 2006; Patriarchi et al., 2018; Buonarati et al., 2019). To test whether such a time course applies to our culture system we monitored clustering of GluA2, which is present in most AMPARs. Although GluA2 clustering became evident 3 min after glutamate treatment, it took 6 min to reach maximum (Figure 6) and lagged behind CaMKII clustering (compare Figure 6M with Figure 5P).

To evaluate whether glutamate-triggered AMPAR clustering reflects an increase in functional AMPAR channels at the postsynaptic site we monitored mEPSCs from cultures treated for 6 min with glutamate followed by a 1 h chase. NMDARs were blocked with MK801 to obtain AMPAR-mediated responses. Similar to the data seen for NMDRs, glutamate treatment nearly doubled the amplitude of AMPAR mEPSCs (Figure 4B). In contrast to NMDRs, the frequency of AMPAR mEPSCs was also substantially increased. This increase in AMPAR mEPSC frequency without concomitant increase in NMDAR mEPSC frequency is indicative of the existence of so-called silent synapses in these neurons at 11DIV, which contain functional NMDARs but not AMPARs (Isaac et al., 1995; Liao et al., 1995; Durand et al., 1996). During development as well as during LTP these silent synapses obtain functional AMPARs (Isaac et al., 1995; Liao et al., 1995; Durand et al., 1996). This transformation occurs during early development even in the absence of dendritic spines (Durand et al., 1996). Our data indicate that an analogous transformation of silent to fully functional synapses occurs upon brief glutamate receptor stimulation in 11DIV hippocampal cultures. However, glutamate-triggered clustering of AMPARs was not maintained (Figure 6N, right panel). This observation indicates additional signaling mechanisms are required for lasting clustering of AMPARs. Alternatively, or in addition, homeostatic mechanisms might lead to reduction of functional AMPARs if the set point for neuronal activity is exceeded by AMPAR clustering following 5 min glutamate treatment in our 11DIV cultures (Watt et al., 2000; Turrigiano and Nelson, 2004). Also, the increase in NMDAR mEPSC could contribute to suppression of AMPAR mEPSCs (Sutton et al., 2006).

### Ca^2+^ influx through NMDARs triggers clustering of NMDARs, AMPARs, and CaMKII

**FIGURE 7:**
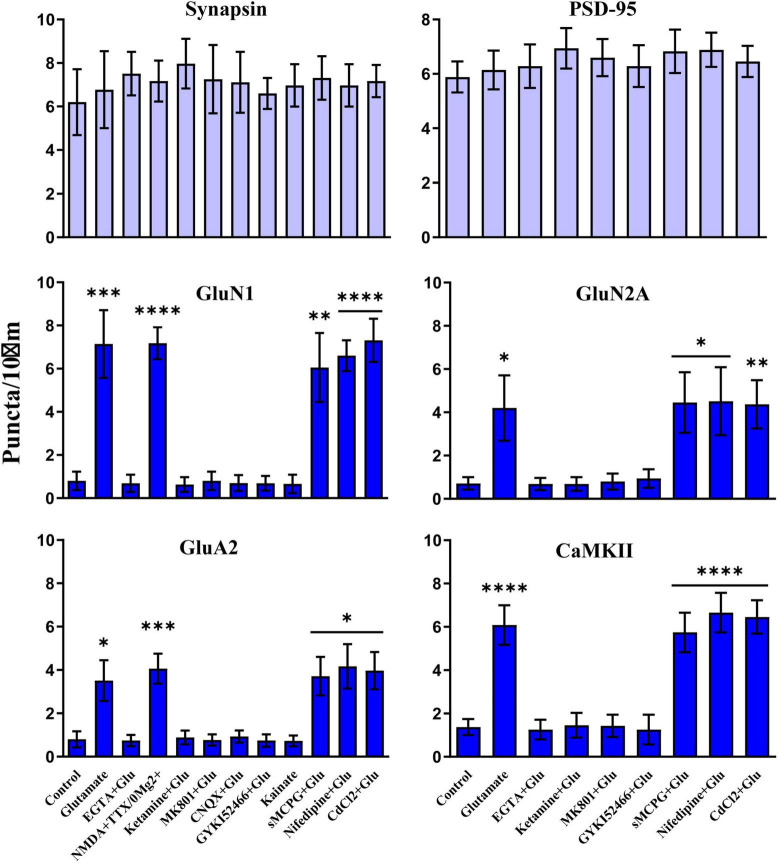
Clustering of glutamate receptors and CaMKII is triggered by Ca^2+^ influx through NMDRs. 11 DIV hippocampal cultures were treated with vehicle (Ctl) or various drugs for 15 min before quantification of total number of synapsin, PSD-95, GluN1, GluN2A, GluA2 and CaMKIIα immunoreactive puncta. Cluster formation of GluN1, GluN2A, GluA2, and CaMKIIα is stimulated by glutamate (100 μM) and, as determined for GluN1 and GluA2, NMDA (100 μM; with 1 μM TTX and no Mg^2+^ present) but not kainate (100 μM). Glutamate-induced clustering is blocked by EGTA (1 mM), ketamine (100 μM), MK801 (40 μM), CNQX (20 μM), and, as determined for GluN1 and GluA2, GYKI52466 (30 μM). It is not inhibited by sMCPG (100 μM), nifedipine (10 μM), or CdCl_2_ (50 μM). Synapsin and PSD-95 distributions remain unaltered. Bars indicate means ± SEMs.

We used different glutamate receptor agonists and antagonists to define the signaling mechanisms that underlie glutamate-induced clustering of NMDARs, AMPARs, and CaMKII. All three events showed the same pharmacological profile. Acute removal of extracellular Ca^2+^ with EGTA prevented clustering, indicating that clustering required Ca^2+^ influx (Figure 7). NMDAR activity was necessary as two different antagonists (ketamine and MK801) blocked glutamate-induced clustering. It was sufficient as NMDA in the absence of Mg^2+^ (but with tetrodotoxin present to restrict the stimulus to NMDAR activation and to prevent epileptiform activity) triggered clustering. However, under physiological conditions with Mg^2+^ present glutamate-induced clustering required co-activation of AMPARs. The clustering was completely prevented by CNQX, which fully blocks AMPARs and kainate receptors. Because CNQX can also reduce NMDAR activity (Lester et al., 1989), we also tested the AMPAR selective GYKI52466, which also completely blocked the glutamate-induced clustering of NMDARs, CaMKII, and AMPARs. AMPAR activation was not sufficient to induce clustering, which is indicated not only by the ketamine and MK801 block but also by a lack of clustering upon kainate treatment. Kainate activates AMPA and kainate receptors but not NMDARs. Notably, AMPARs show much less desensitization when stimulated for prolonged time periods with kainate than with glutamate. Kainate application thus leads to stronger overall AMPAR channel activity than glutamate. The general metabotropic glutamate receptor antagonist sMCPG had no effect on clustering nor did Cd^2+^, which blocks all voltage-gated Ca^2+^ channels, or the L-type Ca^2+^ channel blocker nifedipine. We conclude that glutamate induces postsynaptic clustering of NMDARs, AMPARs, and CaMKII by activating Ca^2+^ influx through NMDARs. AMPARs are required to provide sufficient depolarization to relieve the Mg^2+^ block from NMDARs.

To further evaluate the role of NMDARs in postsynaptic NMDAR clustering, we applied the NMDAR co-agonist glycine. This treatment induces chemical LTP by augmenting NMDAR activation induced through spontaneous release of glutamate (Lu et al., 2001). Because this approach enhances only NMDAR activity at postsynaptic but not extrasynaptic sites, it is thought to better mimic endogenous activity of glutamatergic synapses and the resulting postsynaptic glutamate receptor activation (Lu et al., 2001). Analogous to glutamate treatment, application of glycine for 3–5 min (but not 1 min) also triggered the formation of NMDAR puncta as measured with SEP-GluN1 (Supplementary Figure 6).

### Fast, glutamate-induced NMDAR clustering requires binding of the GluN2A c-terminus to PSD-95 but not to rabphilin 3A

Extensive work indicates that binding of the auxiliary AMPAR subunits known as Transmembrane AMPAR Regulatory Proteins (TARPs) to PSD-95 and its homologues PSD-93 and SAP102 mediates postsynaptic anchoring of AMPARs at postsynaptic sites (Chen et al., 2000; El-Husseini et al., 2000; Schnell et al., 2002; Ehrlich and Malinow, 2004; Schluter et al., 2006; Bats et al., 2007; Ehlers et al., 2007; Elias et al., 2008; Hafner et al., 2015; Matt et al., 2018; Buonarati et al., 2019). Binding of the C-termini of GluN2A and 2B subunits to the PDZ domains of PSD-95 and its homologues (Kornau et al., 1995; Niethammer et al., 1996) is also important for postsynaptic NMDAR localization (Li et al., 2003; Losi et al., 2003; Prybylowski et al., 2005; Elias et al., 2008; Bard et al., 2010), although insight into the role of PSD-95 in postsynaptic NMDAR targeting is more limited than for AMPARs. In addition to PDZ interactions, binding of Rabphilin 3A to residues 1349-1389 in the middle of the GluN2A C-terminus has been implicated in its postsynaptic NMDAR localization (Stanic et al., 2015).

To test whether binding of Rabphilin 3A or PSD-95 to GluN2A or GluN2B is important for the fast glutamate-induced GluN1 clustering, we used myristoylated and thereby membrane-permeant peptides that disrupt these interactions. Peptide 1348 and 1371 are peptides that cover residues 1348-1374 and 1371-1396, respectively, in the middle of the cytosolic C-terminus of GluN2A, which constitutes the Rabphilin 3A binding site. Peptide 1348 and 1371 disrupted binding of Rabphilin 3A but not PSD-95 to GluN2A in acute forebrain slices (Supplementary Figure 7). Peptide 1450 is derived from the very end of the C-terminus of GluN2A and analogous to fluorescein-11R-NR2aCT in Lim et al. (2003). This peptide disrupted the interaction between PSD-95 and GluN2A (Supplementary Figure 7) (Lim et al., 2002, 2003). Peptide 1450 but not 1348 or 1371 impaired the glutamate-triggered NMDAR clustering as detected by imaging of SEP-GluN1 and SEP-GluN2A (Supplementary Figure 8). These data implicate the interaction between the GluN2 C-termini and PSD-95 PDZ domains in the glutamate-induced postsynaptic NMDAR accumulation and thereby into the early steps of postsynaptic recruitment and then anchoring of NMDARs. Still, more work is required to define the molecular details of postsynaptic NMDAR accumulation.

### Stimulation of glutamate receptors does not affect total surface expression of GluN1 and GluA2

We performed cross-linking of surface proteins to test whether the glutamate treatment augmented total surface expression of NMDARs or AMPARs independent of their synaptic localization. This analysis suggests that about 40–50% of the total GluN1 and also GluA2 pool is on the cell surface under basal conditions and this ratio was not affected by glutamate treatments that lasted between 1 and 15 min (Supplementary Figure 9).

## Discussion

### Time course of glutamate-induced postsynaptic clustering of NMDARs, CaMKII, and AMPARs

We demonstrate a surprisingly rapid induction of NMDAR clustering at postsynaptic sites in primary hippocampal cultures at 11 DIV upon activation of pre-existing postsynaptic glutamate receptors. Remarkably, these neurons already possessed numerous synaptic structures with enriched presynaptic and postsynaptic marker proteins and synaptic vesicle exocytosis and endocytosis but low levels of glutamate receptors. NMDARs became concentrated at these synaptic structures within 1 min of glutamate or NMDA treatment and within 3 min of glycine application, which boosted the postsynaptic NMDAR activity that is driven by endogenous spontaneous glutamate release. This accumulation was maintained for at least 3 days. It was followed by accumulation of CaMKII within 2–3 min and AMPARs within 3–6 min. As discussed below, this sequel of clustering events is consistent with the importance of NMDARs as binding sites for CaMKII that are required for its postsynaptic clustering and with CaMKII being important for augmenting postsynaptic AMPAR recruitment.

### Trafficking and postsynaptic accumulation of NMDARs and AMPARs

Insertion of glutamate receptors into the plasma membrane and their internalization by endocytosis occurs at sites outside the postsynaptic membrane proper (Passafaro et al., 2001; Blanpied et al., 2002; Petralia et al., 2003; Ashby et al., 2004; Racz et al., 2004; Oh et al., 2006; Park et al., 2006; Penn et al., 2017). Glutamate receptor trafficking to the plasma membrane is stimulated under various conditions and especially during various forms of synaptic potentiation including LTP in the hippocampal CA1 area (Lledo et al., 1998; Hayashi et al., 2000; Scott et al., 2001; Malinow and Malenka, 2002; Esteban et al., 2003; Park et al., 2004; Oh et al., 2006; Penn et al., 2017). However, it is unclear whether the necessary surface insertion of glutamate receptors has to occur immediately prior to or during potentiation (here induced by glutamate application) in a stimulated fashion or further in advance in a constitutive fashion. The need for earlier surface insertion would reflect a housekeeping role of membrane trafficking that provides a basal level of surface receptors sufficient for induction of postsynaptic glutamate receptor clustering upon potentiation. Because activation of AMPARs and NMDARs was required to induce glutamate receptor clustering in the 11DIV hippocampal cultures that were used here (Figure 7), it seems that functional AMPARs and NMDARs should already have been present at the surface. Consistently, mEPSC analysis indicated substantial synaptic current activity for AMPARs and NMDARs before glutamate-triggered clustering (Figure 4). These observations are in agreement with earlier reports that detected AMPARs, NMDARs, or both at nascent synapses (e.g., Gomperts et al., 1998; Rao et al., 1998; Liao et al., 1999; Washbourne et al., 2002).

Surface labeling suggests that the brief glutamate treatment that induced postsynaptic receptor clustering did not significantly elevate total surface expression of NMDARs or AMPARs (Supplementary Figure 9). Yet mini-EPSCs amplitudes of NMDARs and AMPARs increased (Figure 4) in parallel with their postsynaptic clustering in dendritic spines. Accordingly, the newly arrived glutamate receptors were readily stimulated by presynaptically released glutamate and must have been present at the spine surface. These data suggest that glutamate-induced NMDAR and AMPARs clustering was not driven by their acute surface insertion but rather trapping of NMDARs and AMPARs that reach the postsynaptic sites via lateral diffusion. Earlier work already strongly supported a critical role of lateral diffusion of AMPARs in their postsynaptic clustering during regular development as well as LTP (Tovar and Westbrook, 2002; Tardin et al., 2003; Groc et al., 2004; Adesnik et al., 2005; Ashby et al., 2006; Oh et al., 2006; Bats et al., 2007; Ehlers et al., 2007; Penn et al., 2017). AMPARs can sample numerous postsynaptic sites within minutes and are preferentially trapped at postsynaptic sites of synapses that can undergo spontaneous activity and can release glutamate (Ehlers et al., 2007). Their trapping is mediated by binding of the auxiliary AMPAR subunits called TARPs (e.g., stargazin/γ_2_ and the related γ_8_) to PSD-95, PSD-93, and SAP102 (El-Husseini et al., 2000; Schnell et al., 2002; Elias et al., 2006; Schluter et al., 2006; Bats et al., 2007; Kato et al., 2007; Hafner et al., 2015). NMDARs are thought to be more stably anchored at postsynaptic sites than AMPARs but NMDAR trapping is less well understood. However, it is clear by now that they also traffic to postsynaptic sites by lateral diffusion (Tovar and Westbrook, 2002; Groc et al., 2004, 2006) and are anchored at least to some degree by PSD-95, PSD-93, and SAP102 (Li et al., 2003; Losi et al., 2003; Prybylowski et al., 2005; Elias et al., 2008; Bard et al., 2010) although other mechanisms such as interactions with Ephrin B2 receptor (Dalva et al., 2000; Gonzalez-Gonzalez et al., 2023) and Rabphilin 3A (Stanic et al., 2015) are also important. That peptide 1450, which blocks binding of GluN2 subunits to PSD-95, largely prevented glutamate-triggered NMDAR clustering provides important new specific support for the role of NMDAR anchoring that is mediated by PSD-95 or its homologues SAP102 and PSD-93 during early phases of synapse maturation. At the same time peptides 1348 and 1371, which disrupted the interaction of Rabphilin 3A with GluN2A did not prevent glutamate-induced NMDAR targeting. Apparently, during acute stimulation of postsynaptic NMDAR clustering with glutamate, binding of GluN2A to Rabphilin 3A is not absolutely necessary whereas GluN2A binding to PSD-95 is strictly required. The lack of effect of displacement of Rabphilin 3A from GluN2A does not exclude a role of this interaction in synaptogenesis *in vivo*.

### Mechanisms of postsynaptic CaMKII clustering and its role in AMPAR accumulation

Ca^2+^ influx through NMDARs stimulates binding of CaMKII to NMDAR subunits GluN1, GluN2A and GluN2B (Strack and Colbran, 1998; Leonard et al., 1999, 2002; Bayer et al., 2001; Halt et al., 2012; Hell, 2014). That glutamate-induced CaMKII accumulation occurred subsequent to detectable NMDAR clustering is thus consistent with the importance of NMDAR binding for postsynaptic CaMKII clustering (Halt et al., 2012) [reviewed in Hell (2014), Yasuda et al. (2022)].

That the dodecameric CaMKII is by mass the most abundant protein at the postsynaptic site suggested early on a central role for CaMKII in postsynaptic function (Kennedy et al., 1983; Kelly et al., 1984; Lisman et al., 2002; Hell, 2014; Yasuda et al., 2022). In fact, together with NMDARs, CaMKII plays a critical role in postsynaptic AMPAR recruitment during development (Malenka et al., 1989; Malinow et al., 1989; Wu et al., 1996; Petralia et al., 1999; Liao et al., 2001; Thiagarajan et al., 2002; Pratt et al., 2003; Incontro et al., 2018; Tao et al., 2021) and in synaptic plasticity, especially LTP (Collingridge et al., 2004; Malenka and Bear, 2004; Lisman and Hell, 2008; Huganir and Nicoll, 2013; Hell, 2014; Yasuda et al., 2022). Furthermore, LTP is mediated at least to a substantial degree by conversion of silent synapses that contain only functional NMDARs to those that also contain functional AMPARs (Pettit et al., 1994; Isaac et al., 1995; Liao et al., 1995; Malinow and Malenka, 2002; Collingridge et al., 2004; Malenka and Bear, 2004). Notably, Ca^2+^ influx through the NMDAR triggers CaMKII binding to the NMDAR (Strack and Colbran, 1998; Leonard et al., 1999, 2002; Bayer et al., 2001), which is important for synaptic strength (Pi et al., 2010a,b; Incontro et al., 2018; Tao et al., 2021), activity-dependent spine growth (Hamilton et al., 2012), LTP (Barria and Malinow, 2005; Halt et al., 2012), maintenance of LTP (Sanhueza et al., 2011) (see also (Tao et al., 2021)), and memory formation (Halt et al., 2012). In fact, binding of CaMKII to GluN2B *per se* is sufficient to drive LTP (Tullis et al., 2023).

Accordingly, that glutamate-driven postsynaptic AMPAR clustering occurred after NMDAR and subsequent CaMKII clustering is consistent with a crucial role of NMDAR-anchored CaMKII in postsynaptic AMPAR targeting during synaptic development and potentiation. Collectively these findings indicate that activity-driven fast AMPAR clustering during LTP and upon glutamate application to cultures that contain mostly immature synapses is driven by Ca^2+^ influx through NMDARs and subsequent activation of CaMKII. CaMKII, in turn, can augment postsynaptic AMPAR accumulation via phosphorylation of TARPs (Park et al., 2016), which makes the C-termini of TARPs more accessible for binding to PDZ domains (Sumioka et al., 2010; Hafner et al., 2015). At the same time the bulk of the NMDAR - CaMKII interaction is largely transient and mostly required during early phases of LTP induction and memory consolidation (Shen et al., 2000; Bayer et al., 2001; Lee et al., 2009; Halt et al., 2012) [reviewed in Yasuda et al. (2022)].

### Limitations of the study

In earlier work on glutamate-induced formation of AMPAR clusters, NMDAR cluster number did not change (Lissin et al., 1999; Antonova et al., 2001; Wang et al., 2005). However, in all three studies the hippocampal cultures were clearly more mature than our cultures. The most important evidence for this notion is that in these studies both, NMDAR and AMPAR clusters were very obvious and numerous. Accordingly, synapses appeared to be fully mature and well populated with NMDARs.

In mature synapses, AMPARs are clustered in so-called nanodomains that are juxtaposed to the glutamate release sites for their effective activation (MacGillavry et al., 2013; Nair et al., 2013; Tang et al., 2016; Biederer et al., 2017; Lisman, 2017). A similar nanodomain arrangement is also emerging for NMDARs (Hruska et al., 2018; Kellermayer et al., 2018). Our analysis is based on epifluorescence microscopy, which clearly shows fast glutamate-induced glutamate receptor clustering at postsynaptic sites. However, this analysis cannot directly show their clustering in nanodomains. Defining the glutamate-induced clustering of glutamate receptors on the nanoscale level will be a difficult future quest because defining the exact number of glutamate receptors and their spatial arrangement in such nanodomains is difficult to deduct from the signals that can be obtained from superresolution microscopic methods and thus remains a challenge. Accordingly, there is still a lot of debate on the exact nature of detectable nanodomains for AMPARs and especially NMDARs.

However, the increase in mEPSC amplitudes in parallel with immunofluorescent signals for both, NMDARs and AMPARs provides a clear indication that both receptor types were recruited to such functional nanodomains. At the same time, mEPSC amplitudes only doubled in magnitude, which seems at odds with the quasi all-or-none effect with respect to the appearance of the synaptic NMDAR and AMPAR clusters when there are nearly no clusters detectable before glutamate treatment reflective of an at minimum fivefold increase in synaptic clusters. All these earlier and current findings can be explained by the assumption of the preexistence of NMDAR and AMPAR nanoclusters that are below the detection threshold of epifluorescence microscopy at the immature synapses in 11DIV neurons with glutamate inducing strong accumulation of NMDARs and AMPARs throughout dendritic spines. This accumulation in spines then drives the formation of more or larger nanodomains that are effectively activated by glutamate release. Yet, the remarkably strong increase in synaptic NMDAR and AMPAR immunoreactivity suggests that these receptors are not only forming new nanodomains but also populate the space surrounding them within spines, perhaps for future potentiation of synaptic strength, which leads to the strong increase in fluorescent signals following glutamate treatment. Another aspect important to consider is that many mEPSC events are not discernable from noise, which was in our hands about 10 pA. This factor is especially striking for NMDAR mEPSCs because their amplitudes are much smaller than AMPAR mEPSCs. The reason for this difference is that the number of NMDARs at postsynaptic site is much lower than for AMPARs and that their slow openings occur over a longer time period than the much faster openings of AMPARs. Consistent with these considerations is that the distribution of mEPSC amplitudes for each condition shows a peak with a sharp and often abrupt rise near the 10 pA level (Figure 4 subpanels Ae, Ag, Be, Bg, Cc, Ce, Dc, De). This distribution again suggests that a number of events are below the 10 pA level for both AMPAR and NMDAR mEPSCs. These considerations also explain the difference in mEPSC frequency for AMPARs versus NMDARs.

The surface cross-linking experiments in Supplementary Figure 9 indicate that under basal conditions about half of GluN1 and GluA2 in 11 DIV hippocampal neurons are inside neurons and half on the cell surface. This ratio did not change for either GluN1 or GluA2 upon glutamate treatment. As discussed above, these findings suggest that the glutamate treatment - induced postsynaptic accumulation of NMDARs and AMPARs is mostly based on their lateral diffusion within the plasma membrane and their surface insertion was not substantially stimulated and likely did not provide a large portion of the newly recruited glutamate receptors. However, it cannot be ruled out that a small pool of NMDARs or AMPARs was inserted into the plasma membrane upon glutamate application near postsynaptic sites that could have provided a substantial portion of the newly accumulated glutamate receptors but was not large enough to be detected by surface cross-linking, although this method is quite sensitive.

In any case, the increases in postsynaptic staining and in mEPSC amplitude for NMDARs and AMPARs demonstrate that a sizable pool of both receptors newly accumulates upon glutamate treatment at postsynaptic sites. Together with earlier work our immunofluorescent and EPSC data indicate that this accumulation requires later diffusion, either of pre-existing or newly inserted glutamate receptors.

## Conclusion

Most studies on the functional effects of various synaptic proteins are conducted in hippocampal cultures with mature synapses where NMDARs and AMPARs are conspicuously clustered. We found that synaptic structures in younger neurons (11 DIV) already contain a high level of key pre-and postsynaptic components but a low level of NMDARs and AMPARs; a brief period of Ca^2+^ influx through NMDARs leads to a surprisingly rapid but lasting accumulation of NMDARs and subsequent clustering of CaMKII and AMPARs at postsynaptic sites as part of synapse maturation. Thus, glutamate-triggered signaling can contribute to the maturation of synapses by fostering sequential clustering of NMDARs and AMPARs in young neuronal cultures at preformed synaptic structures.

## Experimental procedures

### Animals

All animal procedures strictly followed NIH guidelines and were approved by the UC Davis Institutional Animal Care and Use Committee (IACUC). Mice were housed on a 12h light / dark cycle, with light on from 7am to 7pm.

### Primary cultures of rat hippocampal neurons

Low-density cultures of dissociated hippocampal neurons were prepared as described (Lim et al., 2003; Chen et al., 2008). Briefly, hippocampi from E18 rats (SD, Harlan) were incubated in Hank’s balanced salt solution (HBSS; Invitrogen), containing trypsin (0.03%) at 37°C for 15 min and washed three times with HBSS before dissociating the cells with a fire-polished pasteur pipette. After non-dispersed pieces had settled, cells in the supernatant were spun down (1,100rpm, 200×g, 1 min), resuspended, counted, and plated at a density of 3–6 × 10^3^ cm^–1^ on coverslips (Warner Instruments, Hamden, CT) coated with 0.1% (w/v) poly-L-lysine (Peptides International, Louisville, KY) in Neurobasal medium (Invitrogen) containing 5% FBS (Fetal Bovine Serum, Gibco) and either 0.5 mM glutamine and NS21 supplement (Chen et al., 2008) without any antibiotic (Figures 1–7 and Supplementary Figures 1, 2, 5, 9) or 1% Glutamax™ (Gibco), B27 supplement (ThermoFisher Scientific), and 1 μg/ml gentamicin (Gibco). After 4 h, medium was replaced with serum-free Neurobasal medium supplemented with glutamine and NS21 or Glutamax™, B27, and gentamicin. Cells were maintained at 37°C in a humidified environment of 95% air/5%CO_2_. One third of medium was changed after 4 DIV. For Supplementary Figures 3, 4, 6, 8, 10 μM 5-fluoro-2′-deoxyuridine (Sigma) was added to cultures 3–7 days after plating to suppress the proliferation of non-neuronal cells. For live detection of GluN1 surface insertion, neurons were transfected with SEP-GluN1 at 7 DIV using calcium phosphate and maintained for an additional 4 days (Supplementary Figure 4).

### Glutamate and glycine treatment of hippocampal cultures

Glutamate (100 μM final concentration) or water (vehicle control) was added directly to culture medium from aqueous neutralized stock for 10 s–15 min (Figures 1–7 and Supplementary Figures 1, 2, 9). In some experiments, including all in which glycine was applied, HCs were washed with artificial cerebrospinal fluid (ACSF, 127 mM NaCl, 26 mM NaHCO_3_, 1.2 mM KH_2_PO_4_, 1.9 mM KCl, 2.2 mM CaCl_2_) for 5 min before 1 ml ACSF containing either glutamate (100 μM final concentration) or glycine (200 μM final concentration (Lu et al., 2001)) was added to each dish. Cultures were washed with phosphate-buffered saline (PBS; DPBS, Invitrogen, Carlsbad, CA) before fixation (PBS plus 4% paraformaldehyde and 4% glucose; 15 min; in some cases cultures were fixed for 10 min with thoroughly dehydrated methanol at −20*^o^*C). For all experiments, control and glutamate-treated cultures were from the same preparation and were processed for immunofluorescence in parallel. Pharmacological agents as follows were added 30 min before glutamate. NMDA (100 μM final concentration), kainic acid (100 μM), 6-cyano-7-nitroquinoxaline-2,3-dione (CNQX; 20 μM), (+)-5-methyl-10,11-dihydroxy-5h-dibenzo (a,d)cyclohepten-5,10-imine (MK801; 40 μM), 6,7-dinitroquinoxaline-2,3(1H,4H)-dione (DNQX; 40 μM), 1 μM tetrodotoxin (TTX; 1 μM), picrotoxin (100 μM), and nifedipine (10 μM) were all from Sigma (St. Louis, MO), glutamate (100 μM), CdCl_2_ (50 μM) and EGTA (1 mM) were from Fisher Scientific, and 4-(8-Methyl-9H-1,3-dioxolo[4,5-h][2,3]benzodiazepin-5-yl)-benzenamine hydrochloride (GYKI 52466; 30 μM) and S-α-methyl-4-carboxyphenylglycine ((S)-MCPG; 100 μM) from Tocris Cookson Inc. (Ellisville, MO). Ketamine (100 μM) was from Abbott Laboratories (N. Chicago, IL). Peptide 1348 (residues 1348-1374: LEDSKRSKSLLPDHTSDNPFLHTYGDD) and 1371 (residues 1371—1396: YGDDQRLVIGRCPSDPYKHSLPSQAV) span the Rabphilin 3A binding site on GluN2A and peptide 1450 the C-terminal sequence of GluN2A (residues 1450-1464: NRRVYKKMPSIESDV). All peptides were myristoylated on their amino-terminal end (ChinaPeptides, Shanghai, China) to make them membrane permeant and were added to culture medium for a final concentration of 10 μM.

### Immunocytochemistry and antibodies

Following fixation, cells were washed with PBS, permeabilized with 0.05% Triton X-100 (20 min), blocked (PBS containing 2% glycerol, 0.05M NH_4_Cl, 5% fetal bovine serum, 2% goat serum; 2 h), incubated with primary antibodies (1.5 h at room temperature or overnight at 4*^o^*C), washed, incubated with Alexa 488 or Alexa 568 conjugated secondary antibodies (Molecular Probes, Eugene, OR; goat anti-rabbit and goat anti-mouse diluted 1:200; for VGlut1 staining goat anti-guinea pig was used 1:700; 1 h, room temperature), washed, and mounted in Prolong Gold Antifade mounting media (Molecular Probes, Eugene, OR). Primary antibodies were mouse monoclonal anti-NR1 (1:100; against GluN1) (Leonard et al., 1999); mouse monoclonal anti-CaMKIIα (1:1,000) (Leonard et al., 1999); mouse monoclonal anti-GluR2 (MAB397, Chemicon, 1:200) (Colledge et al., 2003; Steinberg et al., 2006); mouse monoclonal anti-bassoon (clone SAP7F407, 1:1000; Stressgen) (Passafaro et al., 2003; Takao-Rikitsu et al., 2004; Inoue et al., 2006); mouse monoclonal anti-PSD-95 (clone 7E3-1B8, Affinity BioReagents, Goldern, CO, 1:200) (Colledge et al., 2003); rabbit polyclonal affinity-purified anti-synaptophysin (G95, a kind gift from Dr. R. Jahn, 1:8,000) (Kao et al., 1998; Crump et al., 2001), rabbit antiserum against synapsin 1 (G246, 1:1000; a kind gift from Dr. P. DeCamilli) (De Camilli et al., 1983; Fletcher et al., 1994; David et al., 1996); rat N-terminal anti-synaptotagmin I antibodies (604.1 at 1:40 for uptake by synaptic vesicles in live cultures; 604.2 at 1:50 for total synatpotagmin I labeling in fixed cells; a kind gift from Dr. R. Jahn) (Chapman and Jahn, 1994; Rizzoli et al., 2006; Willig et al., 2006); guinea pig antiserum against VGluT1 (Chemicon; 1:3500; kindly provided by Dr. J. Weiner) (Moulder et al., 2004; Zappone and Sloviter, 2004); mouse monoclonal anti-pan-Shank antibody (clone N23B/49, 1:100; developed by Dr. J. Trimmer with support by NINDS/NIMH and obtained through NeuroMab (Davis, CA)); and rabbit polyclonal anti-NR2A (Upstate, Lake placid, NY 1:100) (Li et al., 2003).

### Immunocytochemistry for analyzing GluN1 surface expression.

Neurons were fixed for 5 min with PBS containing 4% paraformaldehyde and 4% sucrose, washed 3 times in PBS and incubated with blocking solution (PBS containing 2% glycerol, 50 mM NH4Cl, 5% FBS, and 2% normal goat serum (Jackson Labs)) for 1 h. Primary antibody (anti-NR1) diluted in blocking solution was applied to neurons 1 h at room temperature. Cells were subsequently fixed for another 5 min and permeabilized with 0.25% Triton X-100 in PBS for 10 min, incubated with an anti-PSD-95 antibody 2 h at room temperature or overnight at 4°C. After three rinses in PBS and a further incubation with the blocking buffer for 30 min, cells were incubated 1 h with the fluorophore-conjugated secondary antibodies. Cells were rinsed three times in PBS, a very short time in water, and mounted onto microscope slides using Prolong Antifade Reagent (Molecular Probes). For controls, cells were incubated with either the primary or secondary antibodies only to exclude bleed-through, cross reactivity, and non-specific staining by secondary antibodies (not illustrated).

### Image acquisition

Fluorescence microscopy was performed using an IX-70 inverted epifluorescence microscope equipped with a 100x, 0.75 NA objective (Olympus), a MAC2002 shutter (Ludle), and fluorescence filter sets (Chroma, Brattleboro, VT) for Alexa 488 (490 nm band-pass excitation, 528 nm long-pass emission), and Alexa 568 (555nm band-pass excitation, 617 nm long-pass emission). Images were acquired with a ORCA II CCD camera (Hamamatsu, Bridgewater, NJ) equipped with frame grabber EDT DV PCI card controlled by Esee software (Inovision, Chapel Hill, NC). For analysis of SEP-GluN1, a cooled C4742-98 CCD camera (Hamamatsu, Bridgewater, NJ) and Metamorph imaging software in 8-bit format was used. The exposure time and gain setting were the same for all samples within a given experiment.

### Image analysis

For quantification, digitally stored images of fluorescent micrographs were processed with a customized MATLAB program utilizing its image processing toolbox as follows. Measurements were performed by different individuals in a double-blinded manner. The microscope exposure time and gain settings were the same for all snapshots for same staining in both control and glutamate treatment groups. To identify pre- and postsynaptic protein clusters, lower-level staining in neurites was removed with the top-hat function of MATLAB using the same arbitrarily chosen threshold for all pictures. To calculate the degree of colocalization of these clusters, a density threshold was placed with the top-hat function as described above. Clusters were counted and treated as colocalized if their spatial overlay was over 20%. All values in figures and text refer to mean ± SEM from the six samples used for each condition.

Following the initial observation of the robust glutamate-induced NMDAR clustering at 11 DIV we preplanned the following sample structure and statistical data analysis for each condition for the immunofluorescence microscopy. We performed for each condition 3 independent experiments, i.e., we used cultures obtained from three dams in different weeks. For each experiment *n* = 6 fields were randomly chosen in 2–3 different cover slips from 6 different neurons that were far apart and had no overlapping dendritic fields (1 field per neuron) and puncta number was quantified for a total *n* = 18. Graphs show mean ± SEM of these *n* = 18 fields. In some supplemental experiments fewer than 3 different cultures were used if indicated in legends.

### Electrophysiology

Pyramidal neurons were identified by their teardrop-shaped somata with apical-like dendrites. Neurons with this morphology make excitatory glutamatergic connections onto other neurons. Custom 8520 Patch Glass (Warner Instruments, Hamden, CT) electrodes were filled with a solution containing the following: 110 mM K-gluconate, 10 mM KCl, 10 mM HEPES, 5 mM EGTA, 3 mM MgATP, 0.5 mM MgGTP, pH 7.35 with KOH, 240–254 mOsm. Tip resistances were 5–10MΩ. The extracellular solution was: 120 mM NaCl, 3 mM KCl, 3 mM CaCl_2_, 2 mM MgCl_2_, 5 mM Glucose, 0.23 mM Na-Pyruvate, 10 mM HEPES, pH 7.35 with NaOH, 260–270 mOsm. Whole-cell voltage-clamp recordings were obtained at room temperature. Liquid junction potentials were determined to be 5–6 mV; voltages were left uncompensated for the junction potentials. Seal resistances were 3–4 GΩ and resting potentials between −50 and −60 mV. Series resistances (Rs) and input resistances (Rin) were continuously monitored throughout data collection. Neurons in which Rs or Rin changed by more than 10% or in which Rs was >20 MΩ were excluded. Signals were recorded using an Axopatch 200B integrating patch-clamp amplifier, interfaced (Digidata 1322A; Axon Instruments, Foster City, CA) with a Pentium-based computer (Intel) that stored the data and provided on-line response display and off-line data analysis. Sampling rate was 10 kHz and lowpass filter frequency was 1 kHz. pClampex and pClampfit 9.01.07 (Axon Instruments) were used to acquire and analyze data. GraphPad Prism version 5.00 for Windows (GraphPad Software, San Diego, CA) was used for the graphing and statistical analysis (t-test).

NMDAR mEPSCs were recorded in 10–12 DIV neurons clamped to −70 mV with picrotoxin (100 μM), DNQX (40 μM), and TTX (1 μM) in the extracellular medium, in which Mg^2+^ was omitted. AMPAR mEPSCs were monitored in the presence of picrotoxin, MK801 (40 μM), and TTX. The peak amplitude for each event was measured as the point in each event that was furthest from the baseline relative to the baseline amplitude. Instantaneous frequencies were calculated as event frequency at the rate of the current event and the previous event (i.e., the reciprocal of the interevent interval). Each single event in each recorded cell was fully analyzed with respect to rise time (10–90%), amplitude, and decay time constants, calculated using pClampfit. Glutamatergic events were discriminated based on their kinetic characteristics which show different decay times of fast AMPAR and slow NMDAR-mediated events: events with a decay time faster than 15 ms were considered as AMPAR-mediated and events with a decay time between 15 to 50 ms as NMDAR-mediated in the respective recordings. Overlapping events or events with poor baselines were excluded.

Mirroring the imaging experiments, for each condition mEPSC recordings were performed in 3 independent experiments from *n* = 6 neurons that were randomly chosen in 2–3 different cover slips. Averages for each neuron were calculated to obtain a total *n* = 18. Graphs show mean ± SEM.

### Statistical analysis

All values in figures and text refer to mean ± S.E. from three independent experiments. Student’s t test was used for comparisons of two groups, and two-way analysis of variance (ANOVA) for comparisons of more than two groups, followed by Bonferroni’s *post hoc* analysis. *p* < 0.05 was considered statistically significant (*p < 0.05; **p < 0.01; ***p < 0.001; ****p < 0.0001 in all figures).

## Data availability statement

The raw data supporting the conclusions of this article will be made available by the authors, without undue reservation.

## Ethics statement

The animal study was approved by the Institutional Animal Care and Use Committees of the University of Iowa and the University of California at Davis. The study was conducted in accordance with the local legislation and institutional requirements.

## Author contributions

YC: Writing – original draft, Visualization, Validation, Methodology, Investigation, Formal analysis, Data curation, Conceptualization. SL: Writing – review and editing, Visualization, Validation, Methodology, Investigation, Formal analysis, Data curation. AJ: Writing – review and editing, Visualization, Data curation. GJ: Writing – review and editing, Visualization, Data curation. IS: Writing – review and editing, Investigation. JU: Writing – review and editing, Visualization, Validation, Methodology, Investigation. JH: Writing – original draft, Supervision, Resources, Project administration, Funding acquisition, Conceptualization. TP: Writing – review and editing, Visualization, Validation, Investigation.
