# Supplementary material for: Rapid sequential clustering of NMDARs, CaMKII, and AMPARs upon activation of NMDARs at developing synapses
Source: Front Synaptic Neurosci. 2024 Apr 10;16:1291262. doi: 10.3389/fnsyn.2024.1291262 (PMC11039796; doi:10.3389/fnsyn.2024.1291262)
Supplement: Supplementary file 1 [file Data_Sheet_1.PDF]

Privileged Communication
Submitted to Frontiers in Synaptic Neurosciences

SUPPLEMENTAL MATERIAL

Rapid Sequential Clustering of NMDARs, CaMKII, and AMPARs upon Activation of NMDARs at Developing Synapses

Yucui Chen[1], Shangming Liu[2,3#], Ariel A. Jacobi[2], Grace Jeng[2], Jason D. Ulrich[1,4], Ivar S. Stein[1,2], Tommaso Patriarchi[2,5], and Johannes W. Hell[1,2 *]

[1]Department of Pharmacology, University of Iowa
Iowa City, IA 52242-1109, USA

[2]Department of Pharmacology, University of California
Davis, CA 95616-8636, USA

[3]Present Address: Department of Histology and Embryology, Shandong University, Jinan, Shandong, 250012, PR China

[4]Present Address: Department of Neurology, Washington University School of Medicine, St. Louis, MO 63110-1010, USA

[5]Present Address: Chemical Neuropharmacology Group, University of Zürich
CH-8057 Zürich, Switzerland

SUPPLEMENTAL EXPERIMENTAL PROCEDURES

Vesicular Uptake of Anti-Synaptotagmin Antibodies

Primary hippocampal cultures 11 DIV were incubated for 5 min with the monoclonal mouse anti-synaptotagmin I antibody 604.1 (1:40 dilution of hybridoma supernatants; generously provided by Dr. R. Jahn, Max-Planck Institute, Goettingen, Germany). This antibody is directed against the N-terminus of synaptotagmin, which resides in the vesicle lumen and is exposed to the extracellular space upon exocytosis. 604.1 is well established for binding to synaptotagmin during the typically brief extracellular exposures of the lumen of synaptic vesicles (e.g., (Dong et al., 2006; Willig et al., 2006)). Antibody incubation occurred in Tyrode solution (124 mM NaCl, 5 mM KCl, 2 mM $CaCl_2$, 1 mM $MgCl_2$, 30 mM glucose and 25 mM HEPES; pH 7.4 with NaOH) at 0°C (positive control for surface labeling with endocytosis blocked; cultures were cooled down for 5 min at room temperature, and 5 min at 8°C before being put on ice) or 37°C (permissive for synaptic vesicle exocytosis and re-uptake). Cells were either quickly rinsed twice with ice-cold PBS or acid stripped before fixation (4% paraformaldehyde, 4% sucrose, 15 min, room temperature). For acid stripping, cultures were quickly washed with ice-cold TBS (150 mM NaCl, 10 mM Tris-Cl, pH 7.4), incubated for 4 min with 0.5 M NaCl in 0.2 M acetic acid on ice, washed twice with ice-cold TBS and once with ice-cold PBS, and fixed as above. For detection of internalized 604.1 cells were permeabilized after fixation with 0.5% Triton X-100 (15 min, room temperature), washed twice with PBS and blocked (PBS containing 2% glycerol, 0.05 M NH_4Cl, 5% fetal bovine serum, 2% goat serum; 4°C, over night). For detection of surface 604.1 only, cells were blocked without permeablization. All cultures were then incubated with Alexa 568 conjugated secondary antibody (Molecular Probes, Eugene, OR; goat anti-mouse diluted 1:200 in blocking solution; 1 h, room temperature in the dark), washed three times with PBS and twice with H_2O (5 min at room temperature each), and mounted in Prolong Gold Antifade mounting media (Molecular Probes, Eugene, OR) before micrographs were taken and processed.

Immunoprecipitation and immunoblotting

Immunoprecipitation and immunoblotting were performed as in (Patriarchi et al., 2016). Briefly, acute forebrain slices containing hippocampus were incubated with vehicle or myristoylated peptides for 15 min at 32°C, homogenized in IP buffer (1% Triton X-100, 150 mM NaCl, 10 mM EDTA, 10 mM EGTA, 10 mM Tris-HCl, pH 7.4) containing the protease inhibitors pepstatin A (1μg/ml), leupeptin (10μg/ml), aprotinin (20μg/ml) and phenylmethylsulphonylfluoride (200 nM, freshly added before homogenization) (Davare et al., 1999; Hall et al., 2007; Hall et al., 2006). Samples were cleared from non-solubilized material by ultracentrifugation (250,000 x g for 30 min) before IP (4 h at 4°C) with antibodies against GluN2A or control IgG (non-specific rabbit IgG). After gel electrophoresis, proteins were transferred onto polyvinyldifluoride membranes, which were blocked (5% dry milk in Tris-buffered saline, TBS), incubation with primary antibodies (5% dry milk in TBS; 1h), washed with 0.05% Tween-20 in TBS, incubation with horseradish peroxidase (HRP) – conjugated protein A (1:10,000 in 5% dry milk in TBS; 1h), and washed as before for 2h. HRP was detected with ECL plus chemiluminescence reagent (GE Healthcare). Several exposures with increasing exposure times were obtained to ensure that signals were in the linear range, as described (Davare and Hell, 2003; Hall et al., 2006). Antibodies used in IP and IB of GluN2A are described in (Leonard and Hell, 1997) and PSD-95 in (Sans et al., 2000). The Rabphilin 3A antibody had been made against the C-terminal part of Rabphilin 3A and was from Aviva Systems Biology (Product number OAAB104299).

Surface Labeling of Glutamate Receptors with BS^3

The membrane-impermeable, homobifunctional cross-linker BS^3 affords specifically cross-linking of surface proteins including glutamate receptors. Cross-linked subunits will not migrate with the speed monomeric subunits do during SDS-PAGE. Cross-linking will thus result in a reduction of monomeric subunits. Monitoring the extent of such a decrease in monomeric subunits by BS^3 is well established for determining changes in surface expression of glutamate

receptors from samples with small amounts of protein as it does not require additional manipulations such as pull down of surface labeled proteins (e.g., (Boudreau and Wolf, 2005; Greger et al., 2002; Grosshans et al., 2002; Hall and Soderling, 1997)). Cross-linking resulted in the appearance of high molecular mass bands reflecting covalent coupling of the glutamate receptor subunits to neighboring polypeptides likely including other glutamate receptor subunits. Such high molecular weight bands at the top of the gel were pronounced for GluA2 but rather weak for GluN1 (see Supplemental Figure 9) likely because most cross-linked NMDAR complexes are not entering the stacking gel.

Primary hippocampal cultures (10-12 DIV) were treated with 100 μM glutamate or vehicle (water) for 1, 6, or 15 min and washed three times in neurobasal medium containing B27, HEPES, and glutamine, incubated for 5 min at 25ºC and 10 min on ice to halt cellular trafficking, and washed three times in ice-cold 150 mM NaCl, 20 mM HEPES (pH 7.0) to remove amino acids and other primary amines. BS^3 (2 mg/mL) (Pierce) was diluted in a freshly prepared 5 mM citrate buffer (pH 5.0) immediately before use to prevent hydrolysis of the N-hydroxysuccinimide ester moiety. Following 1 h incubation of cultures on ice with BS^3, the cross-linking reaction was quenched by washing cells three times in ice-cold 150 mM NaCl, 100 mM glycine, 20 mM HEPES (pH 7.0). Cells were then lysed (1% SDS, 150 mM NaCl, 50 mM Tris, pH 7.4, 1 mM EDTA, 1 μg/ml leupeptin, 2 μg/ml aprotinin, 1 μg/ml pepstatin A, 3.48 μg/ml phenylmethylsulfonyl fluoride). Proteins were separated using a 7.5% SDS-PAGE gel before transferring to PVDF for immunoblotting and film detection by enhanced chemiluminescence.

REFERENCES

Boudreau, A.C., and Wolf, M.E. (2005). Behavioral sensitization to cocaine is associated with increased AMPA receptor surface expression in the nucleus accumbens. J Neurosci *25*, 9144-9151.

Davare, M.A., Dong, F., Rubin, C.S., and Hell, J.W. (1999). The A-kinase anchor protein MAP2B and cAMP-dependent protein kinase are associated with class C L-type calcium channels in neurons. J Biol Chem *274*, 30280-30287.

Davare, M.A., and Hell, J.W. (2003). Increased phosphorylation of the neuronal L-type Ca(2+) channel Ca(v)1.2 during aging. Proceedings of the National Academy of Sciences of the United States of America *100*, 16018-16023.

Dong, M., Yeh, F., Tepp, W.H., Dean, C., Johnson, E.A., Janz, R., and Chapman, E.R. (2006). SV2 is the protein receptor for botulinum neurotoxin A. Science (New York, NY *312*, 592-596.

Greger, I.H., Khatri, L., and Ziff, E.B. (2002). RNA editing at arg607 controls AMPA receptor exit from the endoplasmic reticulum. Neuron *34*, 759-772.

Grosshans, D.R., Clayton, D.A., Coultrap, S.J., and Browning, M.D. (2002). LTP leads to rapid surface expression of NMDA but not AMPA receptors in adult rat CA1. Nature neuroscience *5*, 27-33.

Hall, D.D., Davare, M.A., Shi, M., Allen, M.L., Weisenhaus, M., McKnight, G.S., and Hell, J.W. (2007). Critical role of cAMP-dependent protein kinase anchoring to the L-type calcium channel Cav1.2 via A-kinase anchor protein 150 in neurons. Biochemistry *46*, 1635-1646.

Hall, D.D., Feekes, J.A., Arachchige Don, A.S., Shi, M., Hamid, J., Chen, L., Strack, S., Zamponi, G.W., Horne, M.C., and Hell, J.W. (2006). Binding of protein phosphatase 2A to the L-type calcium channel Cav1.2 next to Ser1928, its main PKA site, is critical for Ser1928 dephosphorylation. Biochemistry *45*, 3448-3459.

Hall, R.A., and Soderling, T.R. (1997). Differential surface expression and phosphorylation of the N-methyl-D-aspartate receptor subunits NR1 and NR2 in cultured hippocampal neurons. J Biol Chem *272*, 4135-4140.

Leonard, A.S., and Hell, J.W. (1997). Cyclic AMP-dependent protein kinase and protein kinase C phosphorylate N-methyl-D-aspartate receptors at different sites. The Journal of biological chemistry *272*, 12107-12115.

Merrill, M.A., Malik, Z., Akyol, Z., Bartos, J.A., Leonard, A.S., Hudmon, A., Shea, M.A., and Hell, J.W. (2007). Displacement of alpha-Actinin from the NMDA Receptor NR1 C0 Domain By Ca(2+)/Calmodulin Promotes CaMKII Binding. Biochemistry *46*, 8485-8497.

Patriarchi, T., Qian, H., Di Biase, V., Malik, Z.A., Chowdhury, D., Price, J.L., Hammes, E.A., Buonarati, O.R., Westenbroek, R.E., Catterall, W.A.*, et al.* (2016). Phosphorylation of Cav1.2 on S1928 Uncouples the L-type Ca2+ Channel from the β2 Adrenergic Receptor. The EMBO journal *35*, 1330-1345.

Sans, N., Petralia, R.S., Wang, Y.X., Blahos, J., 2nd, Hell, J.W., and Wenthold, R.J. (2000). A developmental change in NMDA receptor-associated proteins at hippocampal synapses. J Neurosci *20*, 1260-1271.

Willig, K.I., Rizzoli, S.O., Westphal, V., Jahn, R., and Hell, S.W. (2006). STED microscopy reveals that synaptotagmin remains clustered after synaptic vesicle exocytosis. Nature *440*, 935-939.

Wyszynski, M., Lin, J., Rao, A., Nigh, E., Beggs, A.H., Craig, A.M., and Sheng, M. (1997). Competitive binding of a-actinin and calmodulin to the NMDA receptor. Nature *385*, 439-442.

SUPPLEMENTAL FIGURES

Supplemental Figure 1. Glutamate Induces Fast GluN2A Redistribution in 11 DIV Hippocampal Neurons. Immunofluorescence images of 11 DIV hippocampal cultures (HC) following treatment with vehicle (**A-C**) or 100 μM glutamate for 1 min (**D-F**). GluN2A is smoothly distributed throughout dendrites but short glutamate applications induce robust clustering (left panels; red in overlay in right panels). Bassoon exhibits a punctate distribution along dendrites under both conditions (middle panels; green in overlay in right panels). Quantification of cluster numbers for GluN2A shows a several fold increase in discernable puncta upon 1-15 min glutamate treatment (see Figure 1Q). Most GluN2A puncta after but not before glutamate treatment are colocalized with synapsin puncta (blue triangles in **G**) indicating that most of the newly formed but not preexisting GluN2A clusters are synaptic.

Supplemental Figure 2. Synaptic Vesicles Undergo Exocytosis and Endocytosis in 11 DIV Hippocampal Cultures. 11 DIV HCs were incubated under control conditions (0 min glu; **A-C**) or with glutamate (**D-F**) for 1 min to test whether synaptic vesicles exocytosis/endocytosis is present at 11 DIV and whether it is affected by glutamate treatment. HCs were kept for another 1 h in medium without glutamate. HCs were then either cooled to 0° (**A, D**) or kept at 37°C (**B, C, E, F**) and incubated with the mouse monoclonal antibody 604.1 against the luminal N-terminus of synaptotagmin I for 5 min. HCs were then washed, fixed, permeabilized if indicated, and stained with a secondary fluorescently-labeled anti-mouse antibody. Surface labeling (without acid wash and without permeabilization) at 0°C when endocytosis is blocked yields a robust signal (**A,D**). This result indicates that a substantial amount of synaptotagmin I is exposed at synaptic puncta and that synaptic vesicles undergo exocytosis in these cultures. Cultures kept at 37°C exhibit little surface labeling (i.e., labeling without permeabilization) after acid wash, which removes antibodies from the surface (**C, F;** both micrographs have a pyramidal cell body in the center with multiple well-developed dendrites as visible by background autofluorescence, which is not illustrated here). Most of the dim puncta in control

panels C and F are not along the somata or neurites and reflect non-specific stainings; furthermore, staining intensities of those puncta are much weaker than for the strong puncta reflecting intracellular synaptotagmin in **B** and **E**. Cultures kept at 37°C that underwent both, acid wash and permeabilization with 0.05 % Triton X-100 exhibit robust synaptic synaptotagmin labeling (**B, E**). These results indicate that synaptotagmin is regularly surface exposed as illustrated by surface labeling at 0°C. It is internalized at physiological temperature because synaptotagmin labeling is only strongly detectable after acid wash if cells were also permeabilized at 37°C (compare B with C and E with F). Similar results were obtained in two independent experiments.

Supplemental Figure 3. Detection of Glutamate-Induced Fast Clustering of GluN1 by surface labeling.

11 DIV HCs were washed with ACSF, which did not contain glycine or glutamate, treated with vehicle (**A-C**) or 50 μM glutamate (**D-F**) for 1 min, fixed, surface stained with the anti-GluN1 antibody, which recognized the extracellular portion of GluN1, permeabilized and stained with anti-PSD95. Surface GluN1 showed smooth distribution throughout the dendrites and cell body under control conditions. Glutamate treatment induced rapid clustering of GluN1 (left panels; red in overlay in right panels). PSD-95 has a punctate appearance along the dendrite under all conditions (middle panels; green in overlay in right panels).

(**G**) Quantification of a total of n=12 dendritic fields from 3 independent experiments (**, control vs glu-1min, $p < 0.01$). PSD-95 puncta showed no significant difference between the conditions.

Supplemental Figure 4. Glutamate-Induced Fast Clustering of SEP-tagged NMDARs.

HCs were co-transfected with SEP-GluN1 and SEP-GluN2A at 7 DIV and imaged at 11 DIV after washing with ACSF, which did not contain glycine or glutamate.

(**A-D**) Treatment with 50 µM glutamate changed the initial smooth distribution of the SEP tag signals (A) to a punctated one.

(**E,F**) Magnified view of the area labeled with red rectangles in A and C.

(**G**) Quantification of puncta density from a total of n=12 dendritic fields from 3 independent experiments (**, $p < 0.01$).

Supplemental Figure 5. Synaptic GluN1 Accumulation Becomes Prominent After 15 DIV in HCs.

Immunofluorescence images of untreated HCs at DIV 16 (**A-C**), 17 (**D-F**), and 18 (**G-I**) double labeled for GluN1 (left panels; red in overlay in right panels) and synapsin (middle panels; green in overlay in right panels). GluN1 puncta become more numerous and prominent after 15 DIV as compared to earlier stages (e.g., control treated cultures at 11 DIV in Figure 1 A-C and at 14 DIV in Figure 3A-C). Similar results were obtained in two independent experiments.

Supplemental Figure 6. Glycine Induces NMDAR Redistribution in HC.

(**A-F**) HCs were transfected with SEP-GluN1 and SEP-GluN2A at 7 DIV and observed at 11 DIV after washing with ACSF. The SEP fluorescence is smoothly distributed along the dendrites under vehicle control conditions (A). Treatment with 100 µM glycine for 1, 3, and 5 min, induced progressive increase in puncta density.

(**E, F**) Magnified views of the areas labeled with red rectangles in (A) and (C).

(**G**) Quantification of puncta density from a total of n=10 dendritic fields from 2 independent experiments (**, $p < 0.01$).

Supplemental Figure 7. Characterization of Myristoylated GluN2A Peptides.

Acute forebrain slices were incubated with vehicle (water; lysate samples on left blots and No Peptide control on right blots) or 10 µM each of GluN2A peptides 1348, 1371, or 1450 for 15

min at 32°C and solubilized before IP with non-specific control antibody (IgG) or an antibody against GluN2A before immunoblotting with antibodies against the proteins indicated on the left side. All peptides were myristolyated to make them membrane-permeant. As expected, 1348 and 1371 displaced Rabphilin 3A but not PSD-95 and 1450 displaced PSD-95 but not Rabphilin 3A. Similar results were obtained in 3 other experiments.

Supplemental Figure 8. C-terminal PDZ Interaction but not Rabphilin 3A Binding is Required for Glutamate-induced NMDAR Clustering.
(**A-D**) HCs were transfected with SEP-GluN1 and SEP-GluN2A at 7 DIV and observed at 11 DIV after washing with ACSF. The neurons were treated with vehicle (water) or 10 μM each of GluN2A peptides 1348, 1371, or 1450 for 1 h before treatment with 50 μM glutamate for 1 min. Peptide GluN2A 1450, which disrupts binding of the NMDAR C-termini to PDZ domains and especially to PSD-95 prevented glutamate-induced clustering. The other two peptides, which disrupt binding of rabphilin 3A to the C-terminal portion of GluN2A, did not affect the clustering. (**F**) Quantification of puncta density from a total of n=10 dendritic fields from 2 independent experiments (**, $p < 0.01$).

Supplemental Figure 9. Glutamate Stimulation does not Significantly Alter Total Surface Expression of GluN1 and GluA2. 11 DIV hippocampal cultures were treated with 100 μM glutamate for 1, 6, or 15 minutes or vehicle control for 15 minutes, cooled to 0°C, incubated with BS^3, and extracted with SDS sample buffer before SDS-PAGE and immunoblotting.
A Representative immunoblots probed with antibodies against GluA2, GluN1, or α-actinin-2, which is a cytoskeletal protein that can interact with NMDARs at their cytosolic domains but is not accessible in intact neurons to BS^3 (Merrill et al., 2007; Wyszynski et al., 1997). Signals for monomeric GluA2 and GluN1, were reduced by cross-linking with BS^3 concomitant with the appearance of high molecular mass bands (compare right lane with left lanes). BS^3 did not

affect the total amount of monomeric α-actinin-2 and no high molecular mass band appeared upon BS^3 treatment indicating that cross-linking was restricted to proteins on the cell surface with extracellular domains.

B. Densitometric quantification of immunoblot signals from 3 independent experiments (mean±SEM). None of the glutamate treated conditions showed a significant difference to vehicle treatment as analyzed by one-factor ANOVA with significance set at $p<0.05$. The reduction in monomeric GluN1 and GluA2 by BS^3 by about 50% indicates that at least 50% of NR1 and GluA2 are surface exposed in 11 DIV hippocampal cultures. This number could be higher if cross-linking is not 100% effective.
