# Supplementary figures and images for: Rapid sequential clustering of NMDARs, CaMKII, and AMPARs upon activation of NMDARs at developing synapses
Source: Front Synaptic Neurosci. 2024 Apr 10;16:1291262. doi: 10.3389/fnsyn.2024.1291262 (PMC11039796; doi:10.3389/fnsyn.2024.1291262)

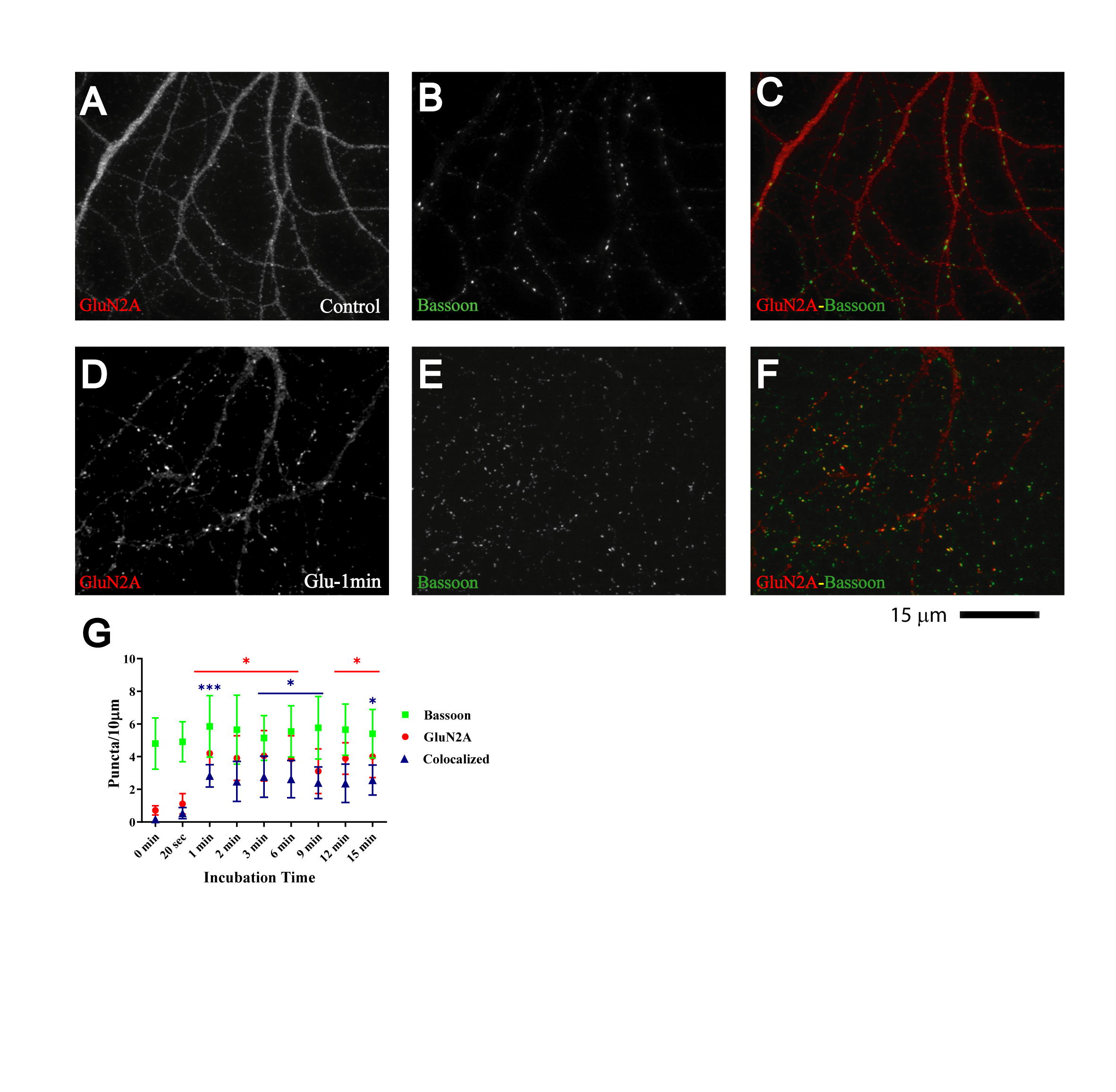

Supplement: Supplementary file 2 [file Image_1.TIF]

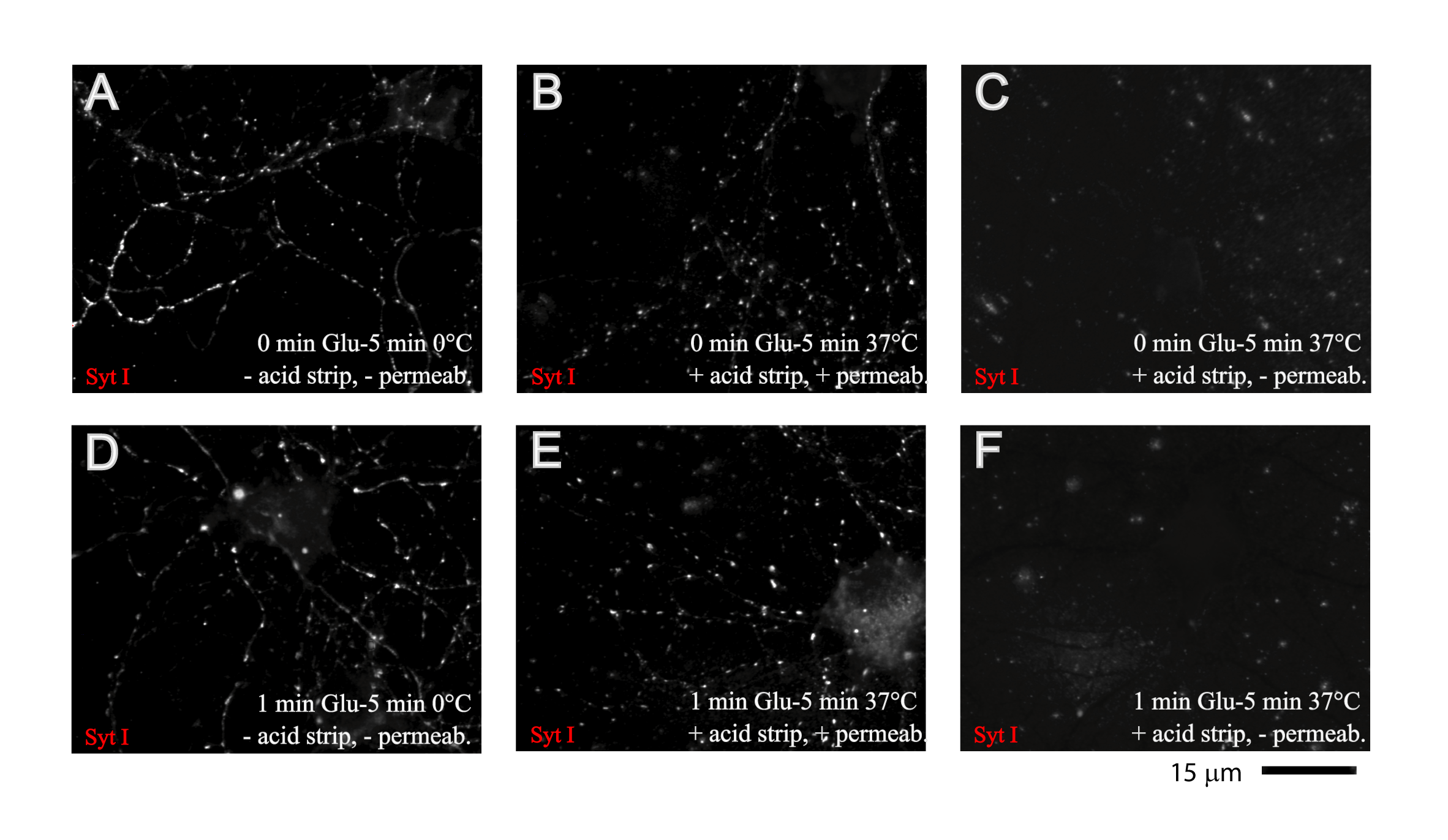

Supplement: Supplementary file 3 [file Image_2.TIF]

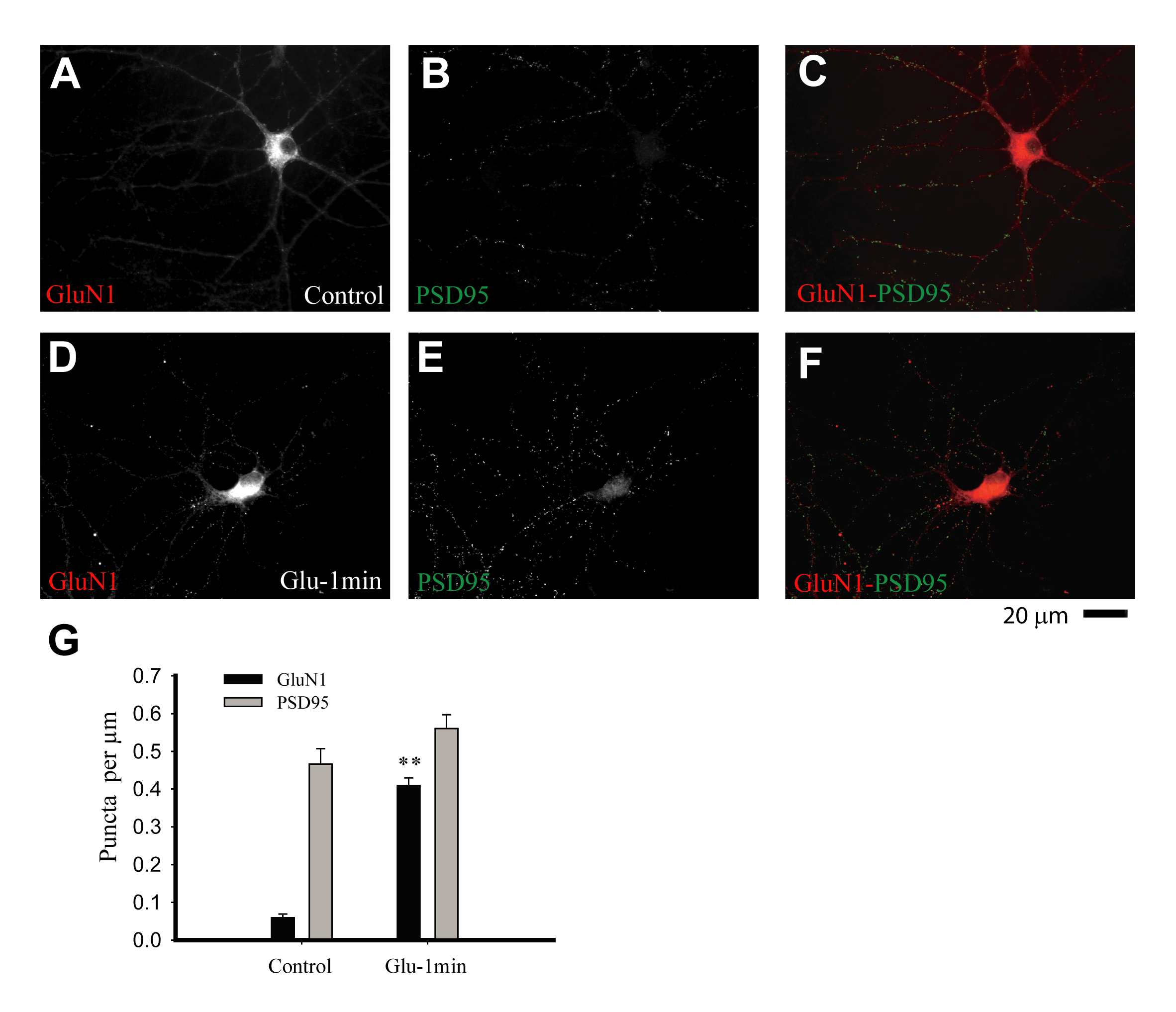

Supplement: Supplementary file 4 [file Image_3.TIF]

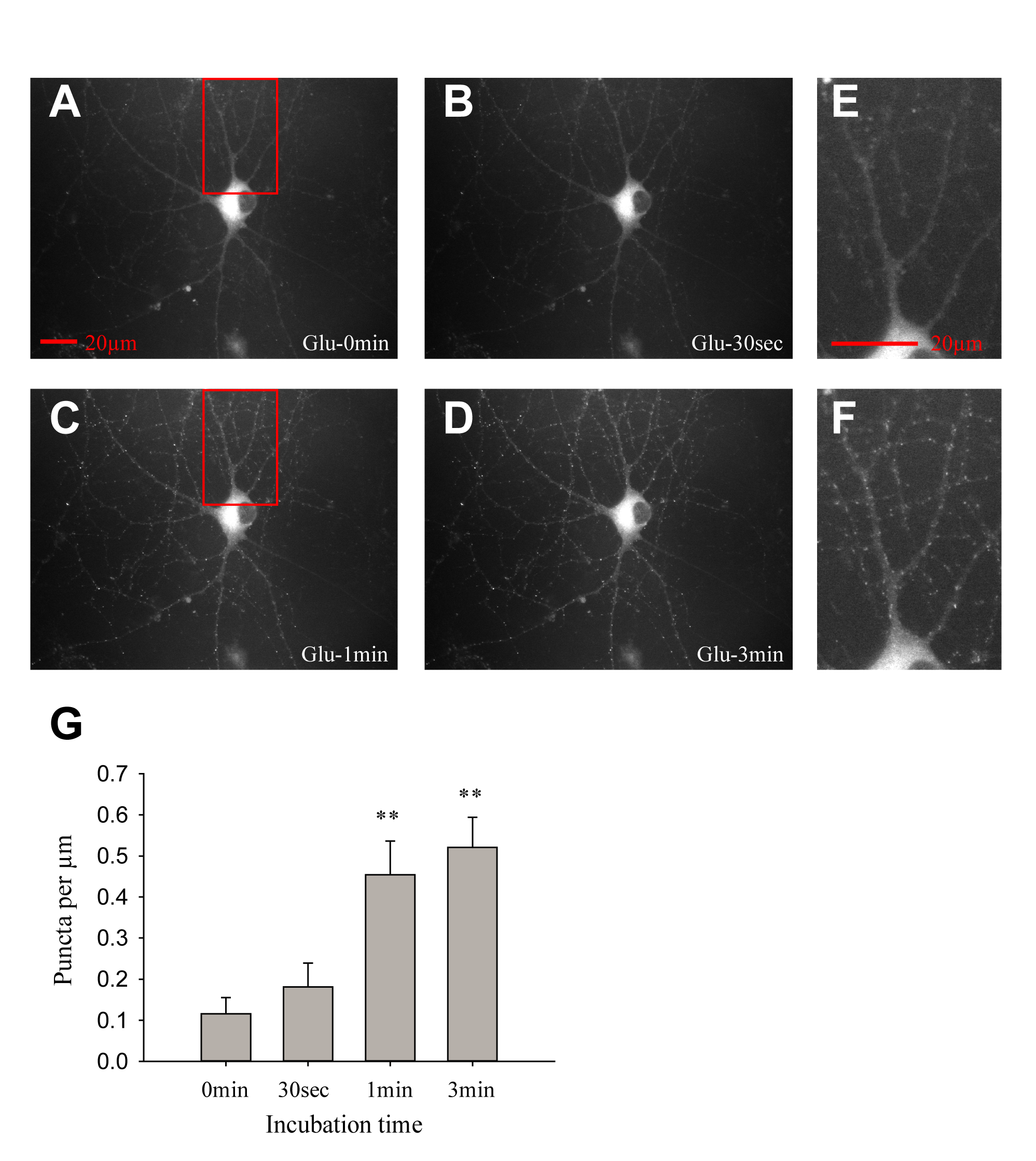

Supplement: Supplementary file 5 [file Image_4.TIF]

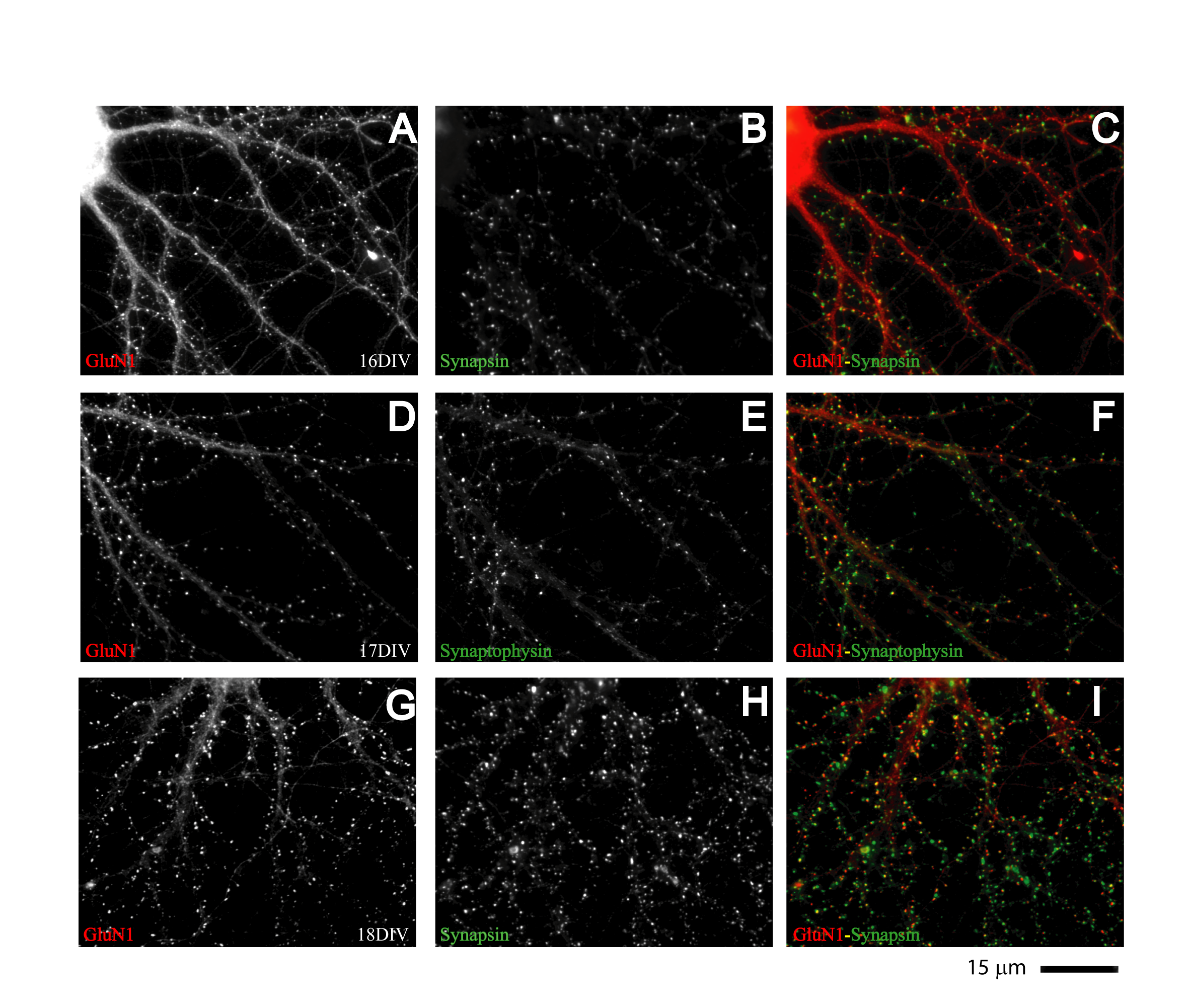

Supplement: Supplementary file 6 [file Image_5.TIF]

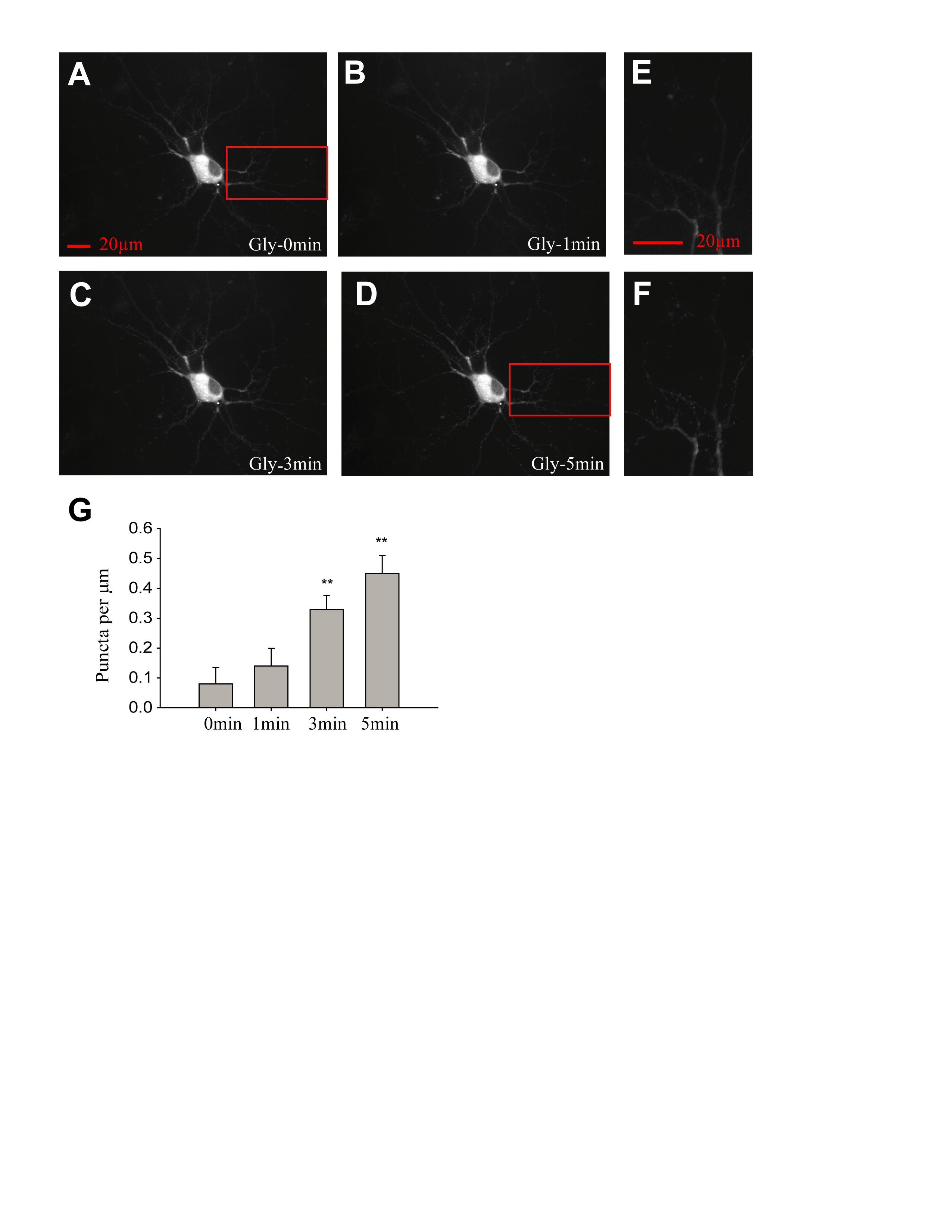

Supplement: Supplementary file 7 [file Image_6.TIF]

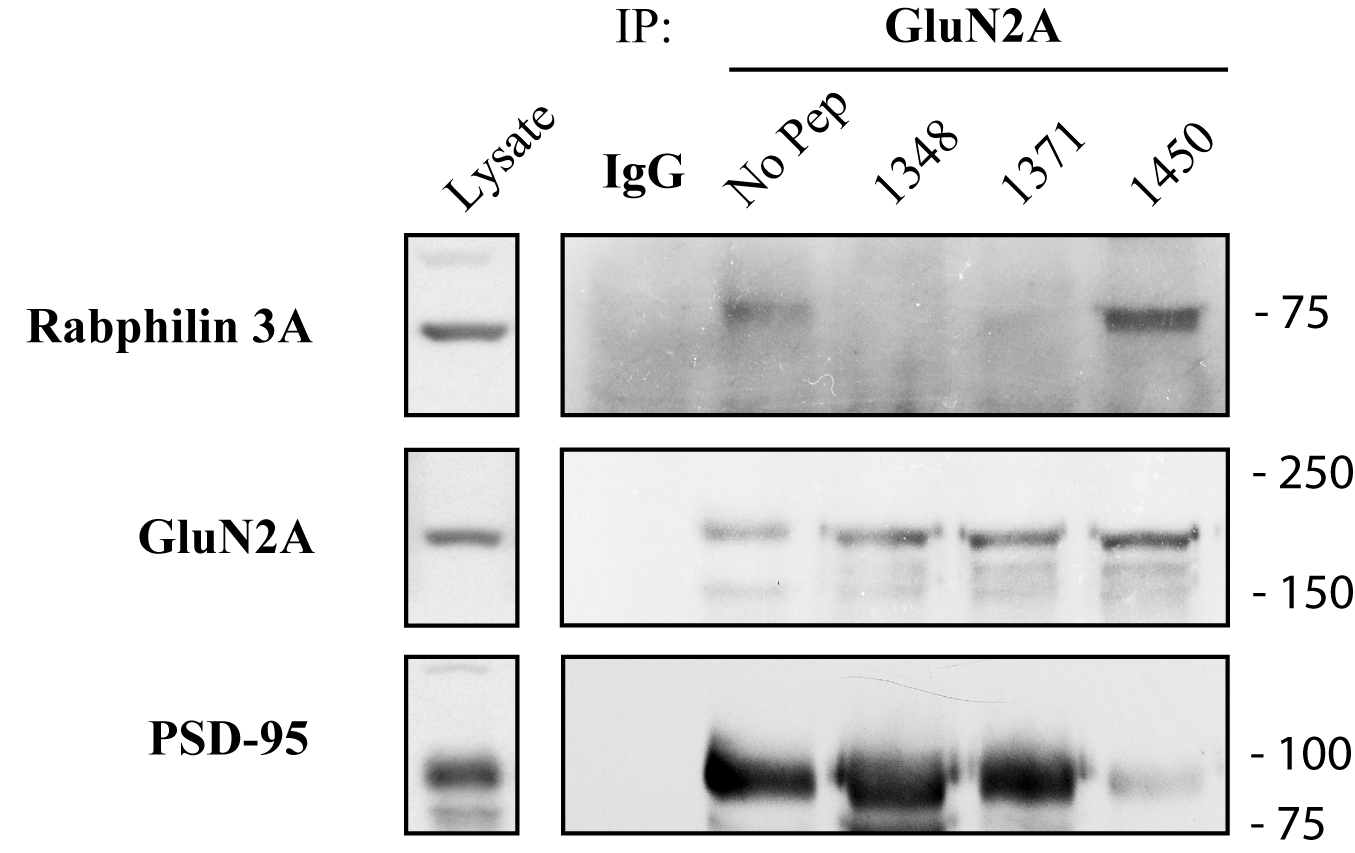

Supplement: Supplementary file 8 [file Image_7.TIF]

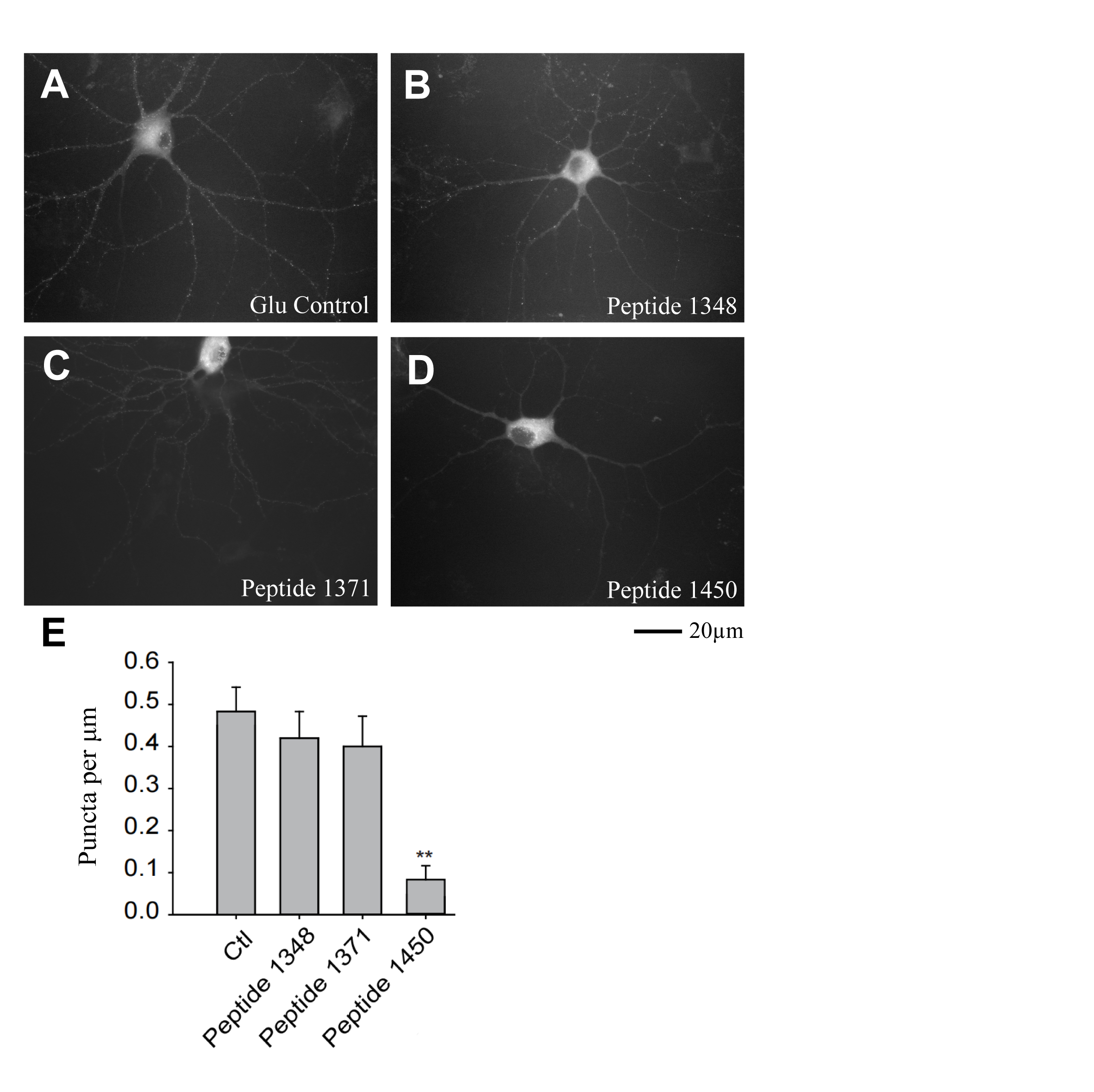

Supplement: Supplementary file 9 [file Image_8.TIF]

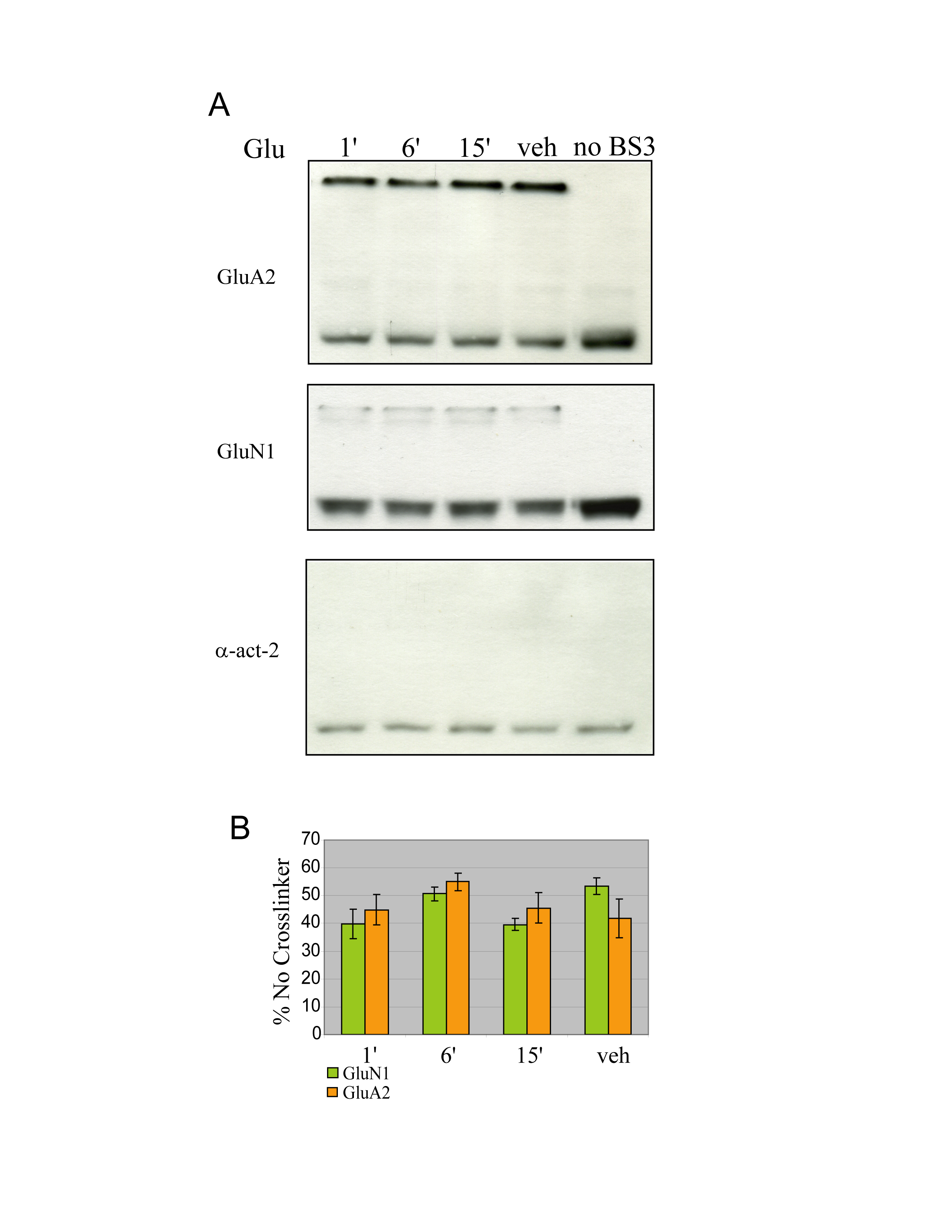

Supplement: Supplementary file 10 [file Image_9.TIF]
